# *Taenia solium* cysticercosis and taeniasis in urban settings: Epidemiological evidence from a health-center based study among people with epilepsy in Dar es Salaam, Tanzania

**DOI:** 10.1371/journal.pntd.0007751

**Published:** 2019-12-06

**Authors:** Veronika Schmidt, Marie-Claire O’Hara, Bernard Ngowi, Karl-Heinz Herbinger, John Noh, Patricia Procell Wilkins, Vivien Richter, Christian Kositz, William Matuja, Andrea Sylvia Winkler

**Affiliations:** 1 Department of Neurology, Center for Global Health, School of Medicine, Technical University of Munich, Munich, Germany; 2 Centre for Global Health, Institute of Health and Society, University of Oslo, Oslo, Norway; 3 Department of Neurology, Elbe Klinikum Stade, Stade, Germany; 4 Muhimbili Medical Research Centre, National Institute for Medical Research (NIMR), Dar es Salaam, Tanzania; 5 College of Health and Allied Sciences, University of Dar es Salaam, Dar es Salaam, Tanzania; 6 Division of Infectious Diseases and Tropical Medicine (DITM), Medical Center of the University of Munich, Ludwig-Maximilians-University Munich, Munich, Germany; 7 Division of Parasitic Diseases and Malaria, Center for Global Health, Centers for Disease Control and Prevention (CDC), Atlanta, Georgia, United States of America; 8 Department of Neurology and Epileptology, Evangelical Hospital Alsterdorf, Hamburg, Germany; 9 Department of Internal Medicine, Kantonsspital St. Gallen, St. Gallen, Schwitzerland; 10 Department of Neurology, Muhimbili University of Health and Allied Sciences, Dar es Salaam, Tanzania; University of Catania, ITALY

## Abstract

In Africa, urbanization is happening faster than ever before which results in new implications for transmission of infectious diseases. For the zoonotic parasite *Taenia solium*, a major cause of acquired epilepsy in endemic countries, the prevalence in urban settings is unknown. The present study investigated epidemiological, neurological, and radiological characteristics of *T*. *solium* cysticercosis and taeniasis (TSCT) in people with epilepsy (PWE) living in Dar es Salaam, Tanzania, one of the fastest growing cities worldwide. A total of 302 PWE were recruited from six health centers in the Kinondoni district of Dar es Salaam. Serological testing for *T*. *solium* cysticercosis-antigen (Ag) and -antibodies (Abs) and for *T*. *solium* taeniasis-Abs was performed in all PWE. In addition, clinical and radiological examinations that included cranial computed tomography (CT) were performed. With questionnaires, demographic data from study populations were collected, and factors associated with TSCT were assessed. Follow-up examinations were conducted in PWE with TSCT. *T*. *solium* cysticercosis-Ag was detected in three (0.99%; 95% CI: 0–2.11%), -Abs in eight (2.65%; 95% CI: 0.84–4.46%), and taeniasis-Abs in five (1.66%; 95% CI: 0.22–3.09%) of 302 PWE. Six PWE (1.99%; 95% CI: 0.41–3.56%) were diagnosed with neurocysticercosis (NCC). This study demonstrates the presence of TSCT in Dar es Salaam, however, NCC was only associated with a few cases of epilepsy. The small fraction of PWE with cysticercosis- and taeniasis-Abs may suggest that active transmission of *T*. *solium* plays only a minor role in Dar es Salaam. A sufficiently powered risk analysis was hampered by the small number of PWE with TSCT; therefore, further studies are required to determine the exact routes of infection and risk behavior of affected individuals.

## Author summary

*Taenia solium* cysticercosis and taeniasis is a zoonotic disease complex which affects thousands of people in sub-Saharan Africa. This parasite has a human-pig life cycle and has been considered a public health problem mainly in rural areas. As African towns and suburbs grow rapidly and disproportionally, adequate infrastructure such as sewage systems and clean water often lack while population density, trade, and travel increase. This may lead to the appearance of parasitic diseases formerly considered `rural´ in urban settings. In this study, we searched for evidence of *T*. *solium* infections in the Kinondoni district of Dar es Salaam, Tanzania. We focused on people with epilepsy (PWE) since epilepsy is one of the most common and severe disorders associated with *T*. *solium* neurocysticercosis and tested all of them serologically for *T*. *solium* cysticercosis and taeniasis. We further investigated neurological and radiological characteristics. Our findings show that in our study area in Dar es Salaam 2.65% of PWE had contracted *T*. *solium* infection at some stage. Neurocysticercosis, as confirmed by neuroimaging, was found only in 1.99% of PWE. This, in combination with the relatively small number of PWE detected with taeniasis antibodies (1.66%), points towards the fact that active transmission of *T*. *solium* seems to play only a minor role in this urban setting, suggesting that infections may mainly be contracted in rural areas. Further large-scale studies are required to investigate the infection pathways and risk behavior related to *T*. *solium* infections within urban areas of sub-Saharan Africa.

## Introduction

*Taenia solium* is a worldwide neglected zoonotic helminth with considerable impact, in endemic countries, on infected humans, animals and the livelihood of their communities. In 2015, the World Health Organization (WHO) Foodborne Disease Burden Epidemiology Reference Group (FERG) published estimates of the global burden of 31 bacteria, viruses, parasites, toxins, and chemicals. *T*. *solium* was identified as the leading cause of deaths from foodborne diseases caused by parasites. In this report, a median of 370,710 global foodborne illnesses, 28,114 deaths and 2,788,426 Disability Adjusted Life Years (DALYs) related to *T*. *solium* were reported [1]. Yet, in many endemic regions, data on the epidemiology and characteristics of the disease complex attributed to *T*. *solium* cysticercosis and taeniasis (TSCT) is lacking, which especially applies to urban areas.

Manifestations of *T*. *solium* in humans can be twofold: taeniasis, the tapeworm infection in the human intestine caused by ingestion of raw and undercooked cysticerci infected pork, and cysticercosis (CC), an infection with larval cysts in multiple organs of pigs and humans. A human tapeworm carrier sheds millions of eggs per day in the stool. Infested human feces are eaten by free-roaming pigs after open defecation or through access to poorly constructed latrines, thereby completing the parasite´s human-pig life cycle. Poor sanitary and handwashing habits represent risk factors for people with taeniasis to infect themselves or other individuals living in close proximity via fecal-oral transmission. After ingestion of eggs larvae will hatch and penetrate the stomach and upper intestine walls, thereby further disseminating in the body, and finally develop into cysts in muscles, subcutaneous or neuronal tissue with a clear preference for the central nervous system tissue (brain and spinal cord), a condition known as neurocysticercosis (NCC) [2–5]. NCC represents a preventable cause of epilepsy and has been specifically identified as a main cause of late onset-epilepsy in developing countries [6–8].

For Tanzania, little data on human CC and NCC is available. A hospital-based cross-sectional study conducted in northern Tanzania in 2006, revealed the proportion of definitive and probable NCC (classified by Del Brutto [9]) for PWE to be 2.4% and 11.3%, respectively [8]. For the Hai district, Tanzania, the seroprevalence of *T*. *solium*-Abs in PWE was 2.8% using the rT24-immunoblot and 1.1% were definitively diagnosed with NCC [10]. In a community-based study conducted in Mbulu district in people without epilepsy (non-PWE), the presence of anticysticercal Abs was reported in 16.3% of 544 individuals using a commercial crude Ag immunoblot [11]. Even less data is available for taeniasis: using microscopy/Kato-Katz or a copro-antigen-enzyme linked immunosorbent assay (copro-Ag-ELISA), the taeniasis prevalence in Tanzania ranged from 0.4% to 1.1% and 0.3% to 5.2%, respectively [12–14]. Presence of adult tapeworm Abs detected by the rES33-immunoblot was found in 4.1% of 820 individuals recruited in Mbeya district and in 0.9% of 213 PWE recruited in the Hai district [10, 15].

To date, research activities on TSCT in sub-Saharan Africa have focused on rural populations, where conditions for maintaining the life cycle of the parasite are favorable [16]. As urban areas in Africa continue to grow disproportionally, conditions for transmission of *T*. *solium* may also be present in many urban and peri-urban settings. The rapid growth of cities often leads to expansion of informal settlements and slums with poor housing, unclean water, the establishment of urban backyard farming, and an inadequate sewage system. Moreover, due to the increase of urban populations and wealth, the demand for pork in towns rises. This goes hand in hand with an increase in informal pig trade and backyard slaughter. Travel of urban populations to and from rural areas also represents a risk factor for the introduction of infectious diseases in towns [17–19]. Rural societies differ from urban societies in terms of demographic patterns, living habits, and co-morbidities. Therefore, data obtained in rural populations should not be applied to urban populations without the generation of evidence. There is clear need for a better understanding of the presence and impact of the TSCT complex in the urban context, as well as of its detailed transmission pathways and specific demographic patterns. To address this gap, we performed a health-center based study in PWE in Dar es Salaam, Tanzania, one of the fastest growing cities in Africa. NCC can be found more often in PWE than in non-PWE [4, 5], which makes this study population ideal to search for evidence of human TSCT in areas where data are not available yet. Hence, the objectives of this study are: 1. to investigate the presence and prevalence of TSCT in PWE in Dar es Salaam, 2. to describe the detailed clinical, serological and radiological characteristics of affected PWE, and 3. to obtain data on associated risk factors for TSCT in PWE living in urban and peri-urban areas.

## Methods

### Settings

**Fig 1:**
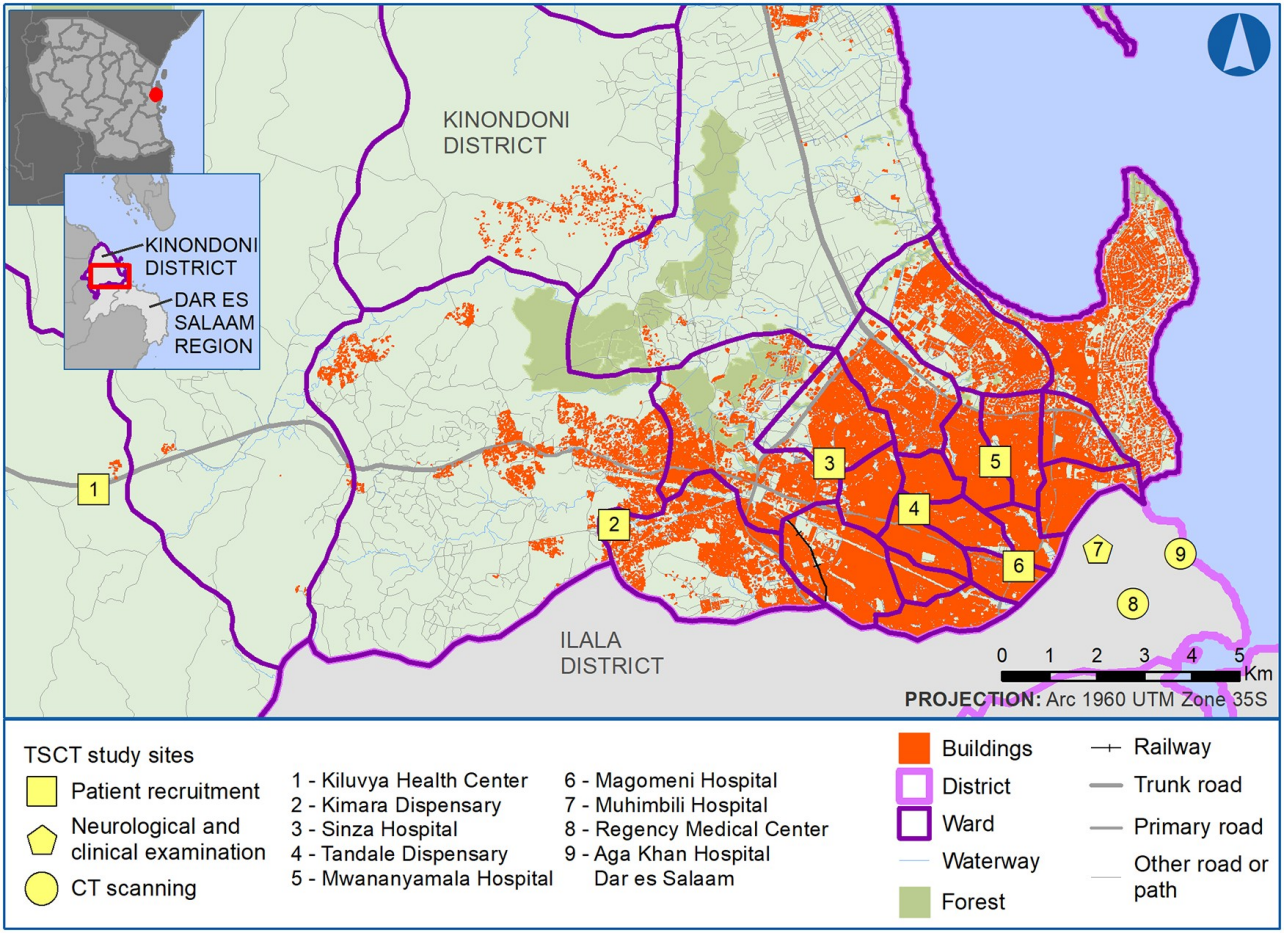
Location of recruitment centers with the distribution of urban and peri-urban areas. Buildings, roads, railways, waterways, and forest data are copyrighted by OpenStreetMap contributors and available from https://www.openstreetmap.org (OpenStreetMap contributors (2016) Planet Dump [Data file from 2016 Aug. 18]). Extract retrieved 2016 Aug. 23 from BBBike, https://download.bbbike.org/osm/). Country, region, district, and ward boundaries are from the GADM database of Global Administrative Areas, v2.8 (November 2015).

This study was conducted in Dar es Salaam, the former capital of Tanzania, with a population of 4.36 million people, which accounts for 10% of the total population according to the 2012 National Census [20]. With an officially reported annual growth rate of 4.39% in 2012 and an estimated population of 5 million by 2020, Dar es Salaam belongs to the ten fastest growing cities in the world [21]. At the time of this study, the city was divided into three municipalities: northern Kinondoni, central Ilala, and southern Temeke. Our study was conducted in Kinondoni municipality, an area of 531 km^2^ with 1.77 million inhabitants in 2012 [22]. The detailed study area is shown in Fig 1.

Administratively, Kinondoni encompasses four divisions and 27 different wards. The original inhabitants of this municipality were the Zaramo and Ndengereko, but urbanization led to a multi-ethnic and multi-religious area. Most likely due to the fast growth of the human population, there is an increasing amount of pigs being kept within the city of Dar es Salaam. The overall annual growth rate of the pig population from 1999 to 2008 was 32.5% [22]. For the agricultural year 2007/2008 a total of 35,479 pigs—kept in 1,937 households (5.7% of all households in the city)—with an average of 18 pigs per pig keeping household—was reported for the city and its suburbs [22]. With its fast urbanization, increasing problems due to insufficient infrastructure (e.g. lack of clean water supply and sewage systems), and a multi-ethnic population, Dar es Salaam has become the epitome of a modern East African city, and therefore was selected as the study site for this survey [17, 23].

### Definitions

PWE were defined as individuals having more than one afebrile seizure unrelated to acute metabolic disorders or withdrawal of drugs and alcohol [24]. Seropositive CC cases were defined as those individuals positive on the lentil lectin purified glycoprotein enzyme-linked immunoelectrotransfer blot (LLGP-EITB), rT24H-immunoblot, or Ag-ELISA test. Seropositive taeniasis cases were defined as individuals positive on the rES33-immunoblot. Individuals with TSCT were defined as those with a positive result in at least one of the four assays. Individuals without TSCT were defined as those with negative results on all serological tests performed. NCC cases were defined as seropositive CC individuals with absolute or highly suggestive NCC lesions detected on computed tomography (CT). Classification in absolute and highly suggestive NCC was performed following the revised criteria by Del Brutto et al. [25]. Viable cysts (active NCC) were defined as cystic lesions (with or without visible scolex) or lesions with ring enhancement. Calcified cysts (inactive NCC) were defined as small hyperdense lesions (calcifications on CT scan) with no sign of ring enhancement [26].

### Enrollment of study participants

Between March 2012 and December 2013 a health center-based recruitment of PWE was performed by a medical officer of Muhimbili University of Health and Allied Sciences (MUHAS) in six governmental health centers and dispensaries located in six wards of the former Kinondoni municipality—Magomeni Hospital (Magomeni), Tandale Dispensary (Tandale), Kiluvya Hospital (Kibamba), Mwananyamala Hospital (Mwananyamala), Sinza Hospital (Sinza) and Kimara Dispensary (Kimara) (Fig 1).

**Fig 2:**
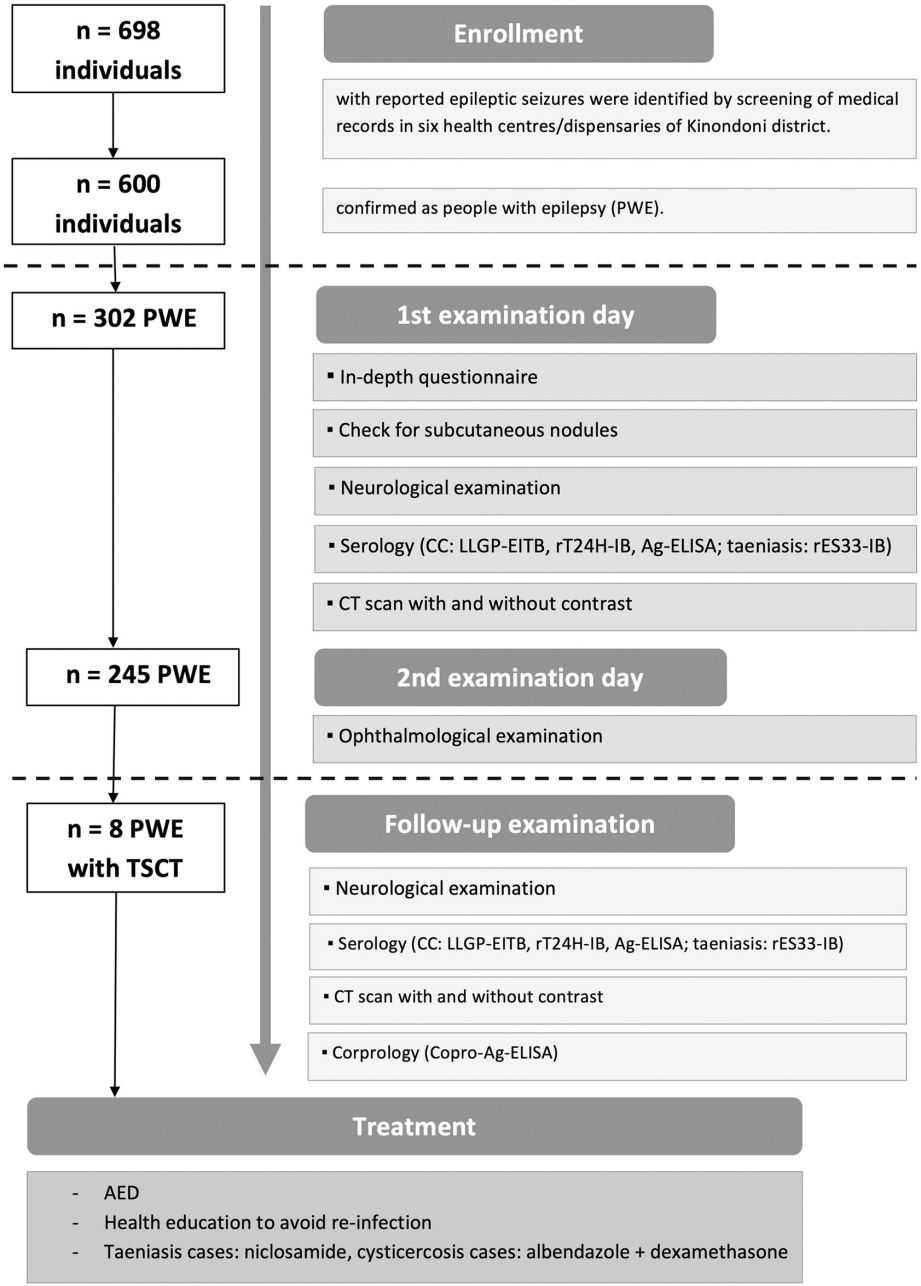
Workflow of recruitment and examinations of people with epilepsy (PWE). CC: cysticercosis; LLGP-EITB: lentil lectin purified glycoprotein enzyme-linked immunoelectrotransfer blot; rT24H-IB: rT24H-immunoblot; Ag: antigen; ELISA: enzyme-linked immunosorbent assay; rES33-IB: rES33-immunoblot; CT: computed tomography; TSCT: *T*. *solium* cysticercosis and taeniasis; AED: anti-epileptic drugs.

For two months, health records were screened retrospectively. A total of 698 individuals with epileptic seizures were identified. Besides a general diagnosis of epilepsy, the following inclusion/exclusion criteria were applied: 1. onset of epilepsy above five years of age to exclude febrile seizures, 2. no relevant history of traumatic brain injuries and 3. no past or present history of any substance abuse. Women who reported pregnant were excluded due to ethical reasons as they would not be able to undergo a CT scan. A total of 600 individuals were confirmed as PWE. Due to financial restrictions, not all identified PWE could be included in this study. Subsequently, 302 PWE were randomly selected and invited by phone to participate and to attend the Hospital of MUHAS. A detailed workflow of enrollment and all examinations can be found in Fig 2.

### Interview and neurological examination

All eligible individuals were invited to the Neurologic Clinic at MUHAS in order to undergo further examinations for two days. During the first examination day a medical doctor in training from the Technical University of Munich, Germany (MCO), together with a health officer re-confirmed inclusion and exclusion criteria by a face-to-face interview. The process was supervised by a Professor of Neurology of MUHAS (WM). Subsequently, each PWE was asked to answer an in-depth questionnaire (see S1 Document). The questionnaire was adapted from a previously validated questionnaire used in community-based epilepsy studies in different African countries and, in addition, addressed socio-demographic features, pork consumption habits as well as hygienic and sanitary practices [27]. In this questionnaire, epileptic seizures were classified as suggested by Winkler et al. [28]. Participants’ mental state, as well as past psychiatric illnesses, were assessed by an experienced neurologist (WM) during the detailed neurological examination. For the diagnosis and classification of psychiatric illnesses, the WHO ICD-10 Classification of Mental and Behavioural Disorders: Diagnostic Criteria for Research was applied [29]. The consent form and questionnaire were written in English, translated to Swahili and back-translated to English by two independent translators. The interview was followed by a standard neurological examination performed by the Professor of Neurology (WM) at the Neurology Unit of the Department of Internal Medicine at MUHAS. If subcutaneous nodules were reported, a clinical check was performed on those subjects.

### Serological testing

For serological testing, a nurse collected 10 ml of whole blood from the cubital vein of each PWE. Blood samples were then immediately stored at +4°C. After one to four hours, a medical technician obtained serum by centrifugation at 3,000 rpm for 5 min. On average, two 2 ml vials of serum were obtained from each PWE, and subsequently transported in cool boxes to the Department of Parasitology and Medical Entomology at MUHAS for further storage at– 20°C. Eight to ten months later, samples were transported to the Centers for Disease Control and Prevention (CDC) in Atlanta, USA, for analyses. Two tests were performed to detect CC-specific Abs (LLGP-EITB and rT24H-immunoblot) and one test each to detect CC-specific Ag (Ag-ELISA), and taeniasis-specific Abs (rES33-immunoblot). LLGP-EITB is an enzyme-linked immunoelectrotransfer blot that detects CC-specific Abs to any of the seven glycoproteins [30–32]. The sensitivity and specificity for the LLGP-EITB is 96%, and 97% with more than two brain cysts. In particular, one of the seven diagnostic bands—the glycoprotein (Gp) 50 band—was reported to potentially cross-react with other parasitic infections such as *T*. *saginata*, *Echinococcus granulosus*, and *Schistosoma ssp*. [32–34]. The rT24H-test is an immunoblot that detects CC-specific Abs to a *T*. *solium* recombinant Ag with a reported sensitivity of 99% and specificity of 100% [32, 35]. Cross-reactions are described with *Entamoeba histolytica*, *Hymenolepis nana* and *Schistosoma ssp*. [35]. The Ag-ELISA that detects *Taenia solium*.-Ag in serum is a monoclonal Abs (B158/B60 Abs) capture-based ELISA and was performed following a modified protocol as described in Dorny et al. [36]. This test has a sensitivity of 90% and a specificity of 98% for the detection of active *T*. *solium* infection. To the current state, it cannot be completely ruled out that, this test may cross-react with other *Taenia ssp*., such as *T*. *saginata*. The rES33-test is an immunoblot that detects adult *T*. *solium* specific-Abs using a recombinant protein derived from the excretory-secretory proteins of the adult tapeworms with a sensitivity of 99% and a specificity of 99.7% [37, 38]. Cross-reactions with *E*. *granulosus* and *S*. *mansonii* were reported in some formats using this Ag [39]. Abs to recombinant peptides, rT24H and rES33 were assessed in the same test [32]. All test results were blinded and then interpreted independently by two scientists (VS and JN) with extensive experience in reading these *T*. *solium* in-house tests. For quality assurance, each positive test was repeated and 15 randomly selected negative samples were also re-analyzed.

### Neuroimaging

After blood collection at MUHAS, PWE were transported by car to external sites for CT scans. Two privately run health facilities with CT scanners in Dar es Salaam were selected for this study due to easy accessibility and high-quality equipment: imaging for 242 PWE was performed at the Regency Medical Center using a 64 slice Philips CT scanner (Philips, Best, The Netherlands). The standard imaging protocol included a non-enhanced and a contrast-enhanced axial scan of the neurocranium with 64 slices and 1 mm slice thickness, with 5 mm slice reconstruction for the cerebrum and 3 mm for the cerebellum. Due to technical problems with this CT scanner, 56 PWE had to be transferred to the Aga Khan Hospital, Dar es Salaam, where the same imaging protocol was performed on a GE CT scanner. CT scan images performed at Aga Khan Hospital were evaluated by radiologists on site and images performed at Regency Medical Center were transferred by telemedicine to radiologists in India for evaluation. Thereafter, all images were stored as hard copies and transferred to the Technical University Munich, Germany, where they were evaluated by a radiologist (VR) and a neurologist experienced in NCC diagnoses (ASW), both of whom were blinded to the clinical diagnosis and the original reporting. One PWE had a CT scan performed within eight months and three patients within one month prior to this study (two at Regency Medical Center and two at Aga Khan Hospital). Records of these former scans were used for evaluation to avoid unnecessary exposure to radiation. CT scans were evaluated for the presence of NCC (viable cysts or calcifications, number of lesions) or any other anomaly.

### Ophthalmological examination

On their second examination day, 245 of 302 PWE (who agreed to participate and presented at the clinic) received an ophthalmological examination by a senior ophthalmologist at the Department of Ophthalmology, MUHAS, including checks for ocular symptoms, measurements of intraocular pressure and visual acuity, fundoscopy and checks for visible cysts under the eyelids, the anterior chamber and other parts of the eye.

### Follow-up of PWE with TSCT

All PWE tested positive to at least one of the serological TSCT tests were asked to return to MUHAS for follow-up examinations and treatment. A second neurological examination was performed by the Professor of Neurology at the Department of Internal Medicine (WM) eight to ten months after the first examination. Due to the delay, which was caused by logistic difficulties, all seropositive PWE were re-invited to a second CT scan shortly before treatment at the Department of Radiology, Aga Khan Hospital. In addition, a second blood sample (10 ml from each individual) was taken at the Department of Internal Medicine, MUHAS. Taeniasis-Abs positive patients were treated with niclosamide 2 g as a starting dosage followed by 1g daily for six days. Active NCC cases were given albendazole 15 mg/kg/day for seven days combined with dexamethasone 24 mg/day. All PWE found positive for TSCT received intensive public health education regarding potential auto-infection and deworming to reduce the risk of a new infection. PWE received symptomatic therapy until serological *T*. *solium* test results were available and decisions on further therapeutic steps could be made. Serum samples were again sent to CDC Atlanta for testing using the same tests as described above. In addition, all seropositive PWE were asked to provide three stool samples (at least 50 ml each) for copro-Ag-ELISA testing before treatment, which was also performed at the CDC Atlanta following a protocol described previously by Guezala et al. This test is described to be species-specific and not to cross-react with *T*. *saginata* [40].

### Data analysis

All data were entered in Excel 2010 (Microsoft, Redmond, WA). All the statistical tests were carried out by EPI INFO 3.3.2. (CDC, Atlanta, GA, USA), and with R statistical software (R version 3.5.2). The descriptive analysis was based on proportions for qualitative variables (Chi-squared test, including Fisher´s exact test) and differences in means for quantitative variables (Mann-Whitney U test). Post hoc tests were performed after categorizing the quantitative variable for the cases in which the Mann-Whitney U test yielded a significant *p*-value. The outcome of these analyses is presented by *p-*values, with the statistical significance defined as *p-*values below 0.05. We considered the Benjamini-Hochberg procedure to evaluate the significance of the individual *p*-values in the context of the multiple testing [41]. Further, univariate and multivariate logistic regression analysis was performed. Ten socio-demographic (sex, age, school education, religion, occupation, period of residency in Dar es Salaam, pork consumption, pork consumption by family, latrine usage, and intake of anthelmintic drugs in the past 12 months) and seven clinical (chronic progressive headaches, age at first seizure, frequency of seizures per month before treatment, type of seizures, motor activity during seizures, aura present before seizures, and psychiatric illness) variables of relevance were included in the univariate analysis. Only those variables showing a *p*-value less or equal to 0.10 were included in the subsequent multivariate logistic modelling. The results of the regression analysis are presented as Odds ratios (OR), including 95% confidence intervals (95% CI), and corresponding *p*-values.

### Ethical statement

Ethical approvals for this study were obtained from the Directorate of Research and Publications, MUHAS, Dar es Salaam (Ref. No.: MU/DRP/REC/Vol.I/36, MU/RP/AEC/Vol.Xii/86 and MU/DRP/AEC/Vol.XVI/91) as well as from the Ethical Committee of Ludwig-Maximilian University (LMU) Munich, Germany. The project proposal and export permits for biological material to the USA were also cleared by the National Institute for Medical Research (NIMR), Tanzania, and a material transfer agreement with the CDC Atlanta was obtained.

Risks and benefits of the diagnostic tests including CT scan were explained to potential participants. Women who were pregnant or reported a missed period were excluded from this study. Following Tanzanian regulations, written informed consent was obtained from participants aged 18 years or more, and oral assent from the patient as well as written consent from a parent/guardian was collected, if patients were under the age of 18 years. In the event of illiteracy, forms were read to the participant and fingerprints were taken. All patients received feedback on the results of their CT scan. In case of any pathological finding that required treatment, patients were referred to the appropriate hospital for treatment.

## Results

### Socio-demographic data and public health characteristics

Three hundred and two PWE were recruited from six health centers and dispensaries of the former Kinondoni district to participate in this study: 108 (35.8%) PWE from Magomeni Hospital, 26 (8.6%) from Tandale Dispensary, 10 (3.3%) from Kiluvya Health Center, 88 (29.1%) from Mwananyamala Hospital, 23 (7.6%) from Sinza Hospital, and 47 (15.6%) from Kimara Dispensary. Locations of recruitment centers are shown in Fig 1.

**Fig 3:**
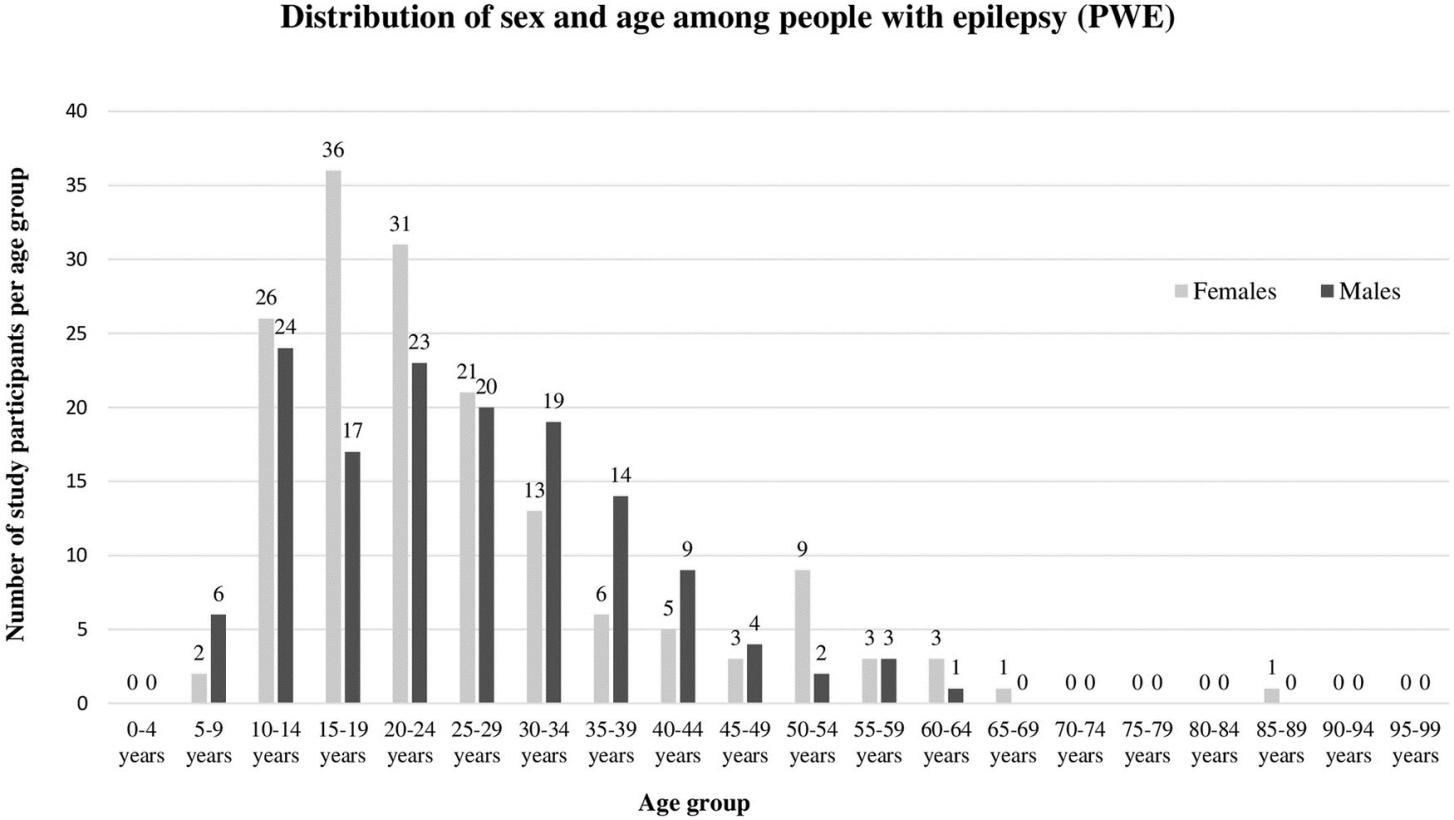
Sex and age distribution of the study population.

The total study population comprised of 160 (53.0%) female PWE. The overall age distribution ranged from 6 to 85 years with a median age of 23 years. PWE were distributed over the chosen age bands as follows: age groups 6–19 years, 20–39 years, 40–59 years, and 60–85 years was 111 (36.8%), 147 (48.7%), 38 (12.6%), and 6 (2.0%) (Fig 3).

The majority (231; 76.5%) of PWE were single with 150 (49.7%) being of Christian faith, followed by 149 (49.3%) who were Muslim. Primary school was attended up to seven years by 248 (83.7%) PWE. Only 24 (8.2%) PWE continued schooling after primary school or went for higher education. No school education was reported by 24 (8.2%) PWE.

The group of PWE infected with TSCT comprised of eight individuals. The median age of PWE with TSCT was 29.5 years and ranged from 18 to 60 years with equal sex distribution. There were more (5; 63.0%) PWE with TSCT found amongst Christians than amongst Muslims (3; 38.0%). In the group of PWE without TSCT, 156 (53.1%) were female. The median age was 23 years and the age distribution ranged from 6–85 years (Table 1). Further socio-demographic characteristics as well as associated factors for TSCT are presented in Table 1. None of these variables were found to be significantly different between PWE with and without TSCT.

**Table 1:** Socio-demographics and associated factors for *T*. *solium* cysticercosis and taeniasis among people with epilepsy.

Variables	People with epilepsy						*p*-value^c^
	seropositive for			seronegative for			
	*T*. *solium* cysticercosis/and taeniasis						
	Number	Proportions (%)^a^	Proportions (%)^b^	Number	Proportions (%)^a^	Proportions (%)^b^	
Total	8	100	-	294	100	-	
Sex							1.00
Female	4	50.00	-	156	53.06	-	
Male	4	50.00	-	138	46.94	-	
Age							0.24
6–19 years	3	37.50	-	108	36.73	-	
20–39 years	3	37.50	-	144	48.98	-	
40–59 years	1	12.50	-	37	12.59	-	
60–85 years	1	12.50	-	5	1.70	-	
School education							0.71
Data known	7	87.50	100	289	98.30	100	
None	0	0	0	24	8.16	8.30	
≤7 years	6	75.00	85.71	242	82.31	83.74	
8–11 years	1	12.50	14.29	23	7.82	7.96	
Religion							0.74
Christian	5	62.50	-	145	49.32	-	
Muslim	3	37.50	-	146	49.66	-	
Other	0	0	-	3	1.02	-	
Occupation							0.83
Data known	8	100	-	284	96.60	100	
Student	0	0	-	93	31.63	32.75	
Business man/woman	3	37.50	-	37	12.59	13.03	
Medical profession	1	12.50	-	6	2.04	2.11	
Gastronomy	0	0	-	3	1.02	1.06	
Other	0	0	-	27	9.18	9.51	
None	4	50.00	-	118	40.13	41.55	
Period of residency in Dar es Salaam							0.92
Data known	8	100	-	285	96.94	100	
3–10 years	1	12.50	-	48	16.33	16.84	
11–20 years	4	50.00	-	113	38.44	39.65	
21–30 years	0	0	-	73	24.83	25.61	
31–85 years	3	37.50	-	51	17.35	17.89	
Pork consumption							0.72
Data known	8	100	-	292	99.32	100	
Yes	5	62.50	-	150	51.02	51.37	
No	3	37.50	-	142	48.30	48.63	
Pork consumption by family							0.26
Data known	7	87.50	100	292	99.32	100	
Yes	6	75.00	85.71	179	60.88	61.30	
No	1	12.50	14.29	113	38.44	38.70	
Latrine usage							0.55
Data known	8	100	-	236	80.27	100	
Always	7	87.50	-	214	72.79	90.68	
Sometimes	1	12.50	-	22	7.48	9.32	
Intake of anthelmintic drug in the past twelve months							0.45
Data known	8	100	-	224	76.19	100	
Yes	1	12.50	-	64	21.77	28.57	
No	7	87.50	-	160	54.42	71.43	

^a^ Proportions among the total number of people with epilepsy, regardless of their data being known or not.

^b^ Proportions among the total number of people with epilepsy, whose data are known.

^c^ Fisher’s exact test was used if at least one cell of the contingency table was below 5.

### Neurological and psychiatric characteristics

Anti-epileptic drugs (AED) were taken by all study participants for whom this information was available (268). The most common AED used was Carbamazepine (126; 41.7%) followed by Phenobarbitone (103; 34.1%). The majority of PWE with TSCT (5/8; 62.5%) and PWE without TSCT (162/232; 69.8%) reported suffering from chronic progressive headaches. A family history of epileptic seizures was reported less frequently in PWE with TSCT compared to those without TSCT (1/7 (14.3%) versus 100/289 (64.6%)), although not significant. Reported age at first seizure was significantly higher in PWE with TSCT (*p* = 0.03): it ranged from 14 to 37 years with a median of 22.5 years, whereas in the group of PWE without TSCT age ranged from 9 to 20 years with a median of 14 years. The 31–85 age group showed a significant correlation with TSCT (*p* = 0.04). The mean frequency of 11 seizures per month before treatment was similarly high in both groups. Type of seizure was significantly correlated with TSCT (*p* = 0.04). Tonic/clonic seizures were the most common presentation and seen at a similar percentage in both groups. Aura before seizures was significantly (*p* = 0.03) more often reported by PWE with TSCT (7/8; 87.5%) than by PWE without TSCT (131/294; 44.6%). Injuries that occurred during seizures were common in both groups. Concomitant psychiatric illness was reported in more than half of the patients in both groups. In PWE with and those without TSCT, psychiatric illness was present in 4 of 7 (57.1%) and 159 of 293 (54.1%), respectively. Among the 163 PWE reporting psychiatric illnesses, 83 (50.9%) suffered from psychotic episodes or behavioral problems, 32 (19.6%) showed mental retardation, 50 (30.7%) reported a cognitive decline, and 14 (8.6%) depression. Two of the four affected PWE with TSCT presented with moderate dementia, one individual reported a cognitive decline for two months only and one severe depression. Detailed neurological and psychiatric characteristics of the study population are shown in Table 2.

**Table 2:** Neurological and psychiatric characteristics of people with epilepsy seropositive and seronegative for *T*. *solium* cysticercosis and taeniasis.

Variables	People with epilepsy						*p-*value^c^	Post hoc *p*-value
	seropositive for			seronegative for				
	*T*. *solium* cysticercosis/and taeniasis							
	Number	Proportions (%)^a^	Proportions (%)^b^	Number	Proportions (%)^a^	Proportions (%)^b^		
Total	8	100	-	294	100	-		
Type of anti-epileptic drug (AED)							1.00	
Data known	8	100	-	260	88.44	100		
Carbamazepine	4	50.00	-	122	41.50	46.92		
Phenobarbitone	3	37.50	-	100	34.01	38.46		
Combination / other AED	1	12.50	-	38	12.93	14.62		
Chronic progressive headaches							0.70	
Data known	8	100	-	232	78.91	100		
Yes	5	62.50	-	162	55.10	69.83		
No	3	37.50	-	70	23.81	30.17		
Family history of seizures							0.43	
Data known	7	100	-	289	98.30	100		
Yes	1	14.29	-	100	34.01	34.60		
No	6	85.71	-	189	64.29	65.40		
Age at first seizure							0.03*	
Data known	8	100	-	291	98.98	100		
5–10 years	0	0	-	100	34.01	34.36		0.06
11–20 years	4	50.00	-	128	43.54	43.99		0.74
21–30 years	1	12.50	-	35	11.90	12.03		1.00
31–85 years	3	37.50	-	28	9.52	9.62		0.04*
Frequency of seizures per month before treatment							0.61	
Data known	8	100	-	284	96.60	100		
0.08–0.9	2	25.00	-	49	16.67	17.25		
1.0–1.9	2	25.00	-	60	20.41	21.13		
2.0–2.9	1	12.50	-	60	20.41	21.13		
3.0–9.9	0	0	-	55	18.71	19.37		
** >**10	3	37.50	-	60	20.41	21.13		
Type of seizures							0.04*	
Gwa	2	25.00	-	171	58.16	-		0.08
Goa	1	12.50	-	44	14.97	-		1.00
Gua	0	0	-	2	0.68	-		1.00
Gfs	3	37.50	-	66	22.45	-		0.39
Gbd	0	0	-	11	3.74	-		1.00
Sp	2	25.00	-	0	0	-		<0.01*
Motor activity during seizures							1.00	
Tonic	0	0	-	28	9.52	-		
Clonic	0	0	-	12	4.08	-		
Tonic / clonic	7	87.50	-	208	70.75	-		
None	1	12.50	-	46	15.65	-		
Aura present before seizures							0.03*	
Yes	7	87.50	-	131	44.56	-		
No	1	12.50	-	163	55.44	-		
Injuries during seizures							0.55	
Data known	8	100	-	293	99.66	100		
Bruise/hematoma	2	25.00	-	37	12.59	12.63		
Burn injury	0	0	-	11	3.74	3.75		
Other	0	0	-	5	1.70	1.71		
None	6	75.00	-	240	81.63	81.91		
Psychiatric illness							1.00	
Data known	7	87.5	100	293	99.66	100		
Yes	4	50.0	57.14	159	54.08	54.27		
No	3	37.5	42.86	134	45.58	45.73		

Gwa: generalized seizures that started within a specific age range (seizures most likely due to idiopathic epilepsy); Goa: generalized seizures that started outside the age range of idiopathic epilepsy but without any obvious sign or history of an underlying cause; Gua: generalized seizures with unknown age of onset; Gfs: generalized seizures with obvious focal neurological signs; Gbd: generalized seizures with more widespread brain damage; Sp: simple partial seizures.

^a^ Proportions among the total number of people with epilepsy, regardless of their data are known or not.

^b^ Proportions among the total number of people with epilepsy, whose data are known.

^c^ Fisher’s exact test was used if at least one cell of the contingency table was below 5.

### Logistic regression models

Univariate logistic regression models were calculated for ten socio-demographic and seven clinical independent variables of relevance, and with TSCT as the dependent variable. These individual models indicate that the age at first seizure (*p* = 0.06) and the presence of an aura before seizures (*p* = 0.04) were significantly associated with TSCT. All other variables presented *p*-values well over the significance limit of 0.10. The results of all individual models are collected in S1 Table.

Variables showing a *p-*value less than 0.10 were utilized to calculate our final model via a multivariate logistic regression. The results of this multivariate analysis confirm an association of PWE being positive for TSCT and the age at which the first seizure occurs (*p <* 0.049; OR 1.05; 95% CI: 1.02–1.08), as well as with having an aura before seizures (*p* = 0.025; OR 14.85; 95% CI: 4.47–49.35).

### *T*. *solium* taeniasis antibodies among PWE

Five PWE positive for taeniasis-Abs out of a total of 302 (1.7%; 95% CI: 0.2–3.1%) were identified during the first and second serological testing. One positive PWE lived in Magomeni, three in Mwananyamala, and one in Kimara ward. At the time of the first serological testing, two out of 302 (0.7%; 95% CI: 0–1.6%) PWE were found positive for taeniasis-Abs. Both PWE were male and 32 and 39 years old. One patient was a businessman and the other was a lab technician. One reported to always use latrines and the other only sometimes. Recruitment centers where PWE with taeniasis-Abs were identified during the first examination are shown in S2 Table.

At the follow-up testing—around eight to ten months later—, both PWE with previously confirmed circulating taeniasis-Abs showed negative results. At this time, three additional (1.0%; 95% CI: 0–2.1%) PWE with TSCT showed positive taeniasis-Abs titers: one was a business man who was 44 years old. He reported to use latrines regularly, and he had taken anthelmintic drugs in the past, but could not specify when and which drug exactly. The two others were Muslim women at the age of 18 years, and both reported to never eat pork, but one reported that the family ate pork. All other (3/5) taeniasis-Abs positive cases identified in this study were Christians reporting to eat pork. The copro-Ag-ELISA, performed at the follow-up examination, was negative in all five PWE identified with circulating taeniasis-Abs (Table 3).

**Table 3:** Serological, neurological and radiological characteristics of people with epilepsy seropositive for *T*. *solium* cysticercosis and taeniasis.

Variables	Patient number (P)							
	P1	P2	P3	P4	P5	P6	P7	P8
Age (years)	19	39	18	27	18	60	44	32
Sex	female	male	female	male	female	female	male	male
Definitive NCC case^a^	no	yes	yes	yes	no	yes	yes	yes
Epileptic seizures since (years)	7	4	4	9	7	1	3	5
Age when epileptic seizures started (years)	13	35	14	18	11	59	41	27
Chronic progressive headaches	yes	no	yes	no	yes	yes	yes	no
Psychiatric symptoms	yes	no	NK	no	yes	yes	yes	no
Laboratory findings (1^st^ examination)								
CC-Ag	n	p	n	n	n	n	p	p
CC-Abs (LLGP)	p	p	p	p	Gp50	p	p	p
CC-Abs (rT24H)	p	p	p	p	n	p	p	p
Taeniasis-Abs	n	p	n	n	n	n	n	p
Computed tomography findings (1^st^ examination)								
Calcifications	no	yes	yes	yes	no	yes	yes	no
Viable cysts	no	yes	no	no	no	no	yes	no
Number of lesions	none	mult	mult	mult	none	mult	mult	none
Perifocal edema	no	yes	no	no	no	no	yes	no
Laboratory findings (2^nd^ examination)^b^								
CC-Ag	n	p	n	n	n	n	p	p
CC-Abs (LLGP)	Gp50	(p)	p	p	p	Gp50	p	n
CC-Abs (rT24H)	n	p	p	p	p	(p)	p	n
Taeniasis-Abs	n	n	(p)	n	(p)	n	p	n
Copro-Ag^c^	n	n	n	n	n	n	n	n
Computed tomography findings (2^nd^ examination)^b^^,^ ^d^								
Calcifications	no	yes	yes	yes	no	yes	yes	yes
Viable cysts	no	no	no	no	no	no	yes	yes

CC: cysticercosis; Ag: antigen; Abs: antibodies; NCC: neurocysticercosis; NK: not known; LLGP: lentil lectin purified glycoprotein enzyme-linked immunoelectrotransfer blot; rT24H: rT24H-immunoblot; p: positive; n: negative; (p): weak positive; Gp50: only the glycoprotein 50-band was detected on the strip; mult: multiple.

^a^ According to the revised diagnostic criteria proposed by Del Brutto [25] and based on both examinations.

^b^ Follow-up after eight to ten months.

^c^ Copro-Ag testing by an enzyme-linked immunosorbent assay could only be performed with the samples of the 2^nd^ examination due to study limitations.

^d^ No information about number and lesions and perifocal edemas were available from the second CT scans, except P8 who had multiple calcifications and viable cysts.

### *T*. *solium* cysticercosis antibodies and antigen

At the time of the first serological testing, eight out of 302 PWE were found positive for CC-Abs (2.7%; 95% CI: 0.8–4.5%), and three of them also showed positive CC-Ag results (1.0%; 95% CI: 0–2.1%; Table 3). Recruitment centers where PWE with CC-Abs and CC-Ag were identified at the first examination are shown in the S2 Table.

At the time of follow-up—eight to ten months later and shortly before treatment—, a second blood sample was taken from those PWE with TSCT. Only four out of eight PWE with initial Abs positive titers showed still a strong positive result in the full LLGP-EITB. One PWE showed a weak positive full LLGP-EITB, two PWE showed only a positive Gp50 band and one sero-reverted.

In three out of eight PWE with TSCT, a positive CC-Ag titer was detected in the first examination and remained positive in the second examination. All three CC-Ag positive PWE showed viable cysts in the CT scan. One CC-Ag positive PWE did not show any lesions in the first CT scans but did on the follow-up scan.

Three PWE with CC-Ab and/or -Ag (P1, P3, P5) were Muslims and five were Christians. Detailed serological Abs and Ag follow-up results in combination with neuroimaging are shown in Table 3.

### *T*. *solium* neurocysticercosis

In this study, a total of six out of 302 (2.0%; 95% CI: 0.4–3.6%) PWE were identified with definitive NCC classified according to the revised criteria of Del Brutto [25]. At the first examinations five (1.7%; 95% CI: 0.2–3.1%) PWE with NCC and at the follow-up one additional NCC patient were diagnosed. Two of eight PWE with TSCT showed no lesions compatible with NCC on CT scan.

Four of six PWE with NCC were males, and the age range was 18–60 years. One PWE with NCC was Muslim and five were Christians. Five individuals reported having suffered from epilepsy for five years and less, and one for nine years. The age at seizure onset ranged from 14 to 59 years. All patients with NCC had multiple lesions. CT scans showed only calcifications in three patients, two patients had calcifications and viable cysts and one patient was diagnosed with only viable cysts. Chronic progressive headache was reported by three of the six PWE with NCC (two of them showed active lesions) and psychiatric symptoms were found in two (moderate dementia and severe depression) of the five patients with available data (one of them with active lesions) (Table 3).

Of the two patients with calcifications and viable cysts, one had multiple scattered calcified nodules bilaterally and an 8 mm temporo-occipital cyst left with minimal eccentric calcification and another 9 mm occipital cyst left, without edema; additionally, mildly enhancing nodules with minimal surrounding edema were found in the frontal lobes bilaterally, the largest measuring 6 mm, and an 8 mm ring-enhancing lesion was seen in the left caudate nucleus. This patient reported no chronic progressive headache and no psychiatric symptoms. The second active NCC patient showed focal white matter edema in the left parietal lobe, a 10 mm cyst with a central isodense nodule in the left caudate nucleus and an 8 mm cyst in the right occipital lobe, additionally, microcalcifications were noted in the temporal lobes and basal ganglia bilaterally. This patient reported chronic progressive headache and severe depression. The PWE with TSCT that was identified with NCC during the follow-up had multiple calcifications and viable cysts. Further radiological details were not available. The patient reported chronic progressive headache but no psychiatric symptoms.

All six NCC patients were CC-Abs positive on both immunoblot, and three were positive on the CC-Ag-ELISA. All PWE with a positive result in the CC-Ag-ELISA had active lesions. Four of six PWE with NCC also had a positive taeniasis-Abs titer.

Detailed serological, neurological and radiological characteristics of PWE with positive TSCT serology with and without NCC at the time of the first examination and the time of follow-up (eight to ten months later) are shown in Table 3.

### Subcutaneous nodules and ocular cysts

There were no subcutaneous nodules found suspicious for *T*. *solium* cysts in any of the 297 PWE, that presented for this examination. Also, the ophthalmological examinations, performed in 245 (81.2%) out of 302 PWE revealed no *T*. *solium* cyst or other *T*. *solium* related changes in the eye or under the eyelids. Fifty-seven PWE did not agree to participate in the ophthalmological examinations or were not presenting at the clinic.

## Discussion

For decades, TSCT has been representing a public health concern in the rural areas of endemic countries. Although Praet et al. demonstrated in 2010 that CC infected pigs reach urban markets of Kinshasa in the Democratic Republic of Congo (DRC) [42], the potential presence of TSCT in urban settings has not yet been explored systematically. Therefore, TSCT prevalence and the impact of this zoonotic disease in urban African settings remains unknown. The current study presents first data on human TSCT in one of the fastest growing cities of Eastern Africa, Dar es Salaam. The core dataset of the study is based on a well-documented demographic, diagnostic, and clinical workup of PWE infected with TSCT. The presence of TSCT in Dar es Salaam was clearly demonstrated in this study. However, prevalence estimates of taeniasis, CC, and NCC in PWE are comparable (taeniasis- and CC-Ab) or lower (CC-Ag and NCC) compared to some estimates reported in rural settings. The proportion of taeniasis-Abs among PWE found in the present study is 1.7%, which is comparable with those recently reported from rural districts in Tanzania, that range from 1.2% (n = 170; non-PWE; by rES33-immunoblot) in southern Tanzania to 4.1% (n = 830; non-PWE; by rES38-immunoblot) in northern Tanzania [17, 43, 44]. Previous estimates of taeniasis prevalence in non-PWE based on copro-Ag-ELISA results mostly vary from 0.1% to 4% in community-based settings [15, 45]. Regarding taeniasis prevalence estimates, it has to be considered that specifically the rES38-immunoblot and most copro-Ag-ELISA protocols used in these studies are known to cross-react with *T*. *saginata*, which might have contributed to some of the higher reported rates. Although the information is still scarce, a recent review revealed that *T*. *saginata* is present in Tanzania and that it is generally widespread in humans and cattle in Eastern Africa [46].

All PWE identified in the present study with a positive taeniasis-Abs titer also have a positive CC-Abs result. Despite the number of PWE with taeniasis-Abs being relatively small (five including results of the serological follow-up), this could potentially point to a high potential of auto-infection. Evidence for the risk of auto-infection has already been demonstrated by Garcia and Del Brutto (1999), who reported that patients with massive brain infections have a higher probability of carrying a tapeworm (11 patients, 82% with tapeworm infection) [47], and by Gilman et al. (2000), who showed that the frequency of taeniasis in NCC patients may reach 15% [48]. However, all PWE with taeniasis-Abs tested negative in the copro-Ag-ELISA. Two of those PWE presented negative Ab results eight to ten months later during the second examination when the copro-Ag ELISA was performed. This sero-reversion could be explained by a transient infection that was cleared and did not result in detectible Ag levels in the stool. However, during the second examination in three formally negative PWE taeniasis-Abs were detected (two showing only a light positive result) whilst the copro-Ag test which was performed at the same time was negative. Besides a recent new exposure, a false positivity due to cross-reacting parasites could potentially explain this discrepancy. Both, the rES33 immunoblot and the copro-Ag-ELISA protocol described by Guezala et al. (2009) have not been reported to cross-react with *T*. *saginata* [38–40]. However, in previous studies using different formats (like a multiantigen print immunoassay) the rES33-antigen showed cross-reactions with *E*. *granulosus* and with *S*. *mansonii* [39], which are both prevalent in Tanzania [49, 50]. The study of the latter was conducted in Dar es Salaam [50]. A microscopic and molecular examination of stool samples might have provided a clearer picture. Unfortunately, they could not be included due to study limitations.

Overall, our taeniasis-Ab results suggest that new *T*. *solium* infections caused by ingestion of infested pork is probably scarce in Dar es Salaam and that active transmission of *T*. *solium* may only play a minor role within the urban environment. The study performed by Praet et al. (2010) in Kinshasa already suggested that highly infected animals are excluded at a certain level in the pig trade chain to urban markets. Although low infected pork may reach urban markets and street kitchens, the infection pressure on exposed humans in urban settings seems to be low [42]. This is also supported by the fact that pigs are mainly kept in confinement in these urban surroundings, with no access to human stool. In the present study, all PWE with TSCT reported a residency in Dar es Salaam of at least six years. However, a detailed travel history was not collected, and therefore, it is not unlikely that *T*. *solium* infections may have been obtained during a trip to endemic rural areas (e.g. for visiting relatives). From a public health perspective, it has to be considered that tapeworm carriers in towns are potentially able to put more people at risk when compared to rural settings. Relevant urban conditions include an increased number of people using one shared pit latrine, increased number of pit latrines of which many are inadequate especially during flooding in wet seasons, small distances between pit latrines and wells, and disposal of feces in plastic bags in slum areas. These conditions may support increased human-to-human transmission and contamination of the environment [51–53]. Given that most NCC patients in this study seem to have been exposed to at least one adult tapeworm it may be useful to routinely examine the stool of NCC patients for *T*. *solium* eggs and provide intensive public health education on prevention of TSCT re-infections.

Interestingly, three out of eight PWE with TSCT identified in this study were businessmen/women and one PWE was a lab technician. It could be that some professions are at a higher risk and need specific attention and access to information for prevention. Moreover, two taeniasis-Ab positive PWE were of Muslim faith who reported never to eat pork. However, one Muslim NCC patient (P3) with a positive taeniasis-Ab result reported that her family consumed pork. This points to *T*. *solium* infections potentially being present in Muslim communities in Tanzania. Therefore, *T*. *solium* infections should not be excluded *a priori* in these communities. For quality assurance all positive tests were repeated in this study; nevertheless, we would like to emphasize that false positivity caused by parasitic cross-reaction cannot be excluded in these cases.

CC-Abs were present in 2.7% PWE. This result is in agreement with a seroprevalence of 2.8% among PWE from a door-to-door study (n = 218) conducted in the Hai district, Tanzania [10], and a seroprevalence of 1.8%, from a study (n = 278) conducted in Ifakara, Tanzania, among PWE [43]. In both cases, studies were performed in rural areas and calculations were based on rT24-immunoblot results.

A large variety of prevalence estimates can be seen in the percentages of CC-Ag among PWE. In the present study, the proportion of 0.99% is comparable with the proportion reported from a case-control study (1.4%; n = 210) conducted in PWE in rural settings in The Gambia [54]. Nevertheless, the majority of studies conducted in rural African communities indicate higher prevalence estimates for CC-Ag among PWE, ranging up to 23.2% as reported from Zambia [55–57]. All these studies, including ours, used the same Ag-ELISA (B158/B60-ELISA) for Ag-testing. It cannot be totally ruled out, that the higher values are the result of other *Taenia ssp*. infections, such as *T*. *saginata*, or different cut-off values used for reading the ELISA. However, in our study Ag-ELISA results fit together well with the neuroimaging results; all CC-Ag positive PWE showed active lesions on CT-scan.

Among our eight PWE identified with positive TSCT serology, six had definitive NCC according to the revised criteria of Del Brutto et al. [25], and the overall proportion of definitive NCC in our study is 1.99%. The review of Bruno et al. (2013) describes median urban NCC prevalence estimates in PWE in Latin America from 1.2% (Brazil) to 5.4% (Peru) based on CT scan and LLGP-EITB results [58], the former close to our results. Also, the proportion of NCC in this report is lower than that from a hospital-based cross-sectional study conducted in northern rural Tanzania in 2006 in 212 PWE, which revealed a proportion of definitive NCC of 2.4% based on Ab-detection and CT scan imaging following the revised diagnostic criteria for NCC by Del Brutto et al. [3, 25]. A cross-sectional, community-based study performed in rural Zambia in 49 PWE, reported a proportion of 4.1% (using CT scan and Ag-ELISA results) [57], and a health center-based study in southern Rwanda reported 7.4% of definitive NCC in 215 PWE based on CT scan and Ab-ELISA results [59]. All three African studies were conducted in rural communities. When comparing prevalence estimates in PWE from previous studies to those in our study, our findings indicate that NCC in Dar es Salaam only affects a very small proportion of PWE. Nonetheless, the presence of NCC cases indicates that both serological and radiological diagnostics, as well as appropriate training of medical staff in *T*. *solium* infections are important for early-case detection and therapy. However, there are currently no serological *T*. *solium* tests available in Tanzanian laboratories and PWE are currently not tested for NCC (Dr. Bernard Ngowi, pers. comm.).

Along with epilepsy, chronic progressive headaches and psychiatric conditions are the main clinical symptoms/signs of NCC [5]. In the present study, out of six PWE with NCC one had moderate dementia and one reported severe depression. Three of six NCC patients reported chronic progressive headaches. Two of those PWE showed active lesions on CT scan. Whether the headaches were due to brain infection or epilepsy itself cannot be ascertained. Most PWE with negative TSCT results also reported headaches.

Seizure types among PWE with NCC are described in the literature to be very heterogenic. This was also confirmed by Singh et al. [60], who reported that 34.6% of PWE with NCC had seizures of any type (*p* < 0.001). In our study, which identified and examined only eight PWE with TSCT, focal seizure types prevailed in PWE with TSCT which was further supported by the fact that PWE with TSCT reported significantly more often an aura (*p* = 0.03), which in itself represents a focal seizure, compared to PWE without TSCT. In our study, the type of seizure was significantly correlated with TSCT (*p* = 0.04). However, results regarding the type of seizure should be interpreted with great caution due to the lack of statistical power and due to the fact that the subgroup `Simple partial seizures´ included only two cases. The univariate logistic analysis that we performed did not show a significant association between the type of seizure and TSCT in PWE (*p* = 0.58). However, in both logistic regression analysis, the univariate (*p* = 0.04) and the multivariate (*p* = 0.049), having an aura before seizures were clearly associated with TSCT. Despite NCC being considered a major cause of late-onset epilepsy in endemic countries, the age group (31–85 years) at seizure onset that was significantly correlated with NCC in the bivariate analysis (*p* = 0.04) and the logistic regression analysis *(p* = 0.025) is still higher than age ranges described in other studies [60–62]. A reason for this could be that the home environment is less contaminated with *T*. *solium* eggs, henceforth infection is acquired in later adult life when eating in public places as well as moving and travel becomes more frequent.

Regarding serology, all six NCC patients were CC-Abs positive, but only three were positive in the CC-Ag-ELISA (all three with active lesions on CT scan). Therefore, our results suggest that in addition to neuroimaging both—CC-Ab and -Ag testing—should be performed for diagnosis in NCC suspected individuals. Interestingly, two PWE with negative rT24H results and no lesions on CT scan showed at the same time a single positive band (Gp50) on the LLGP-EITB, but were clearly CC-Ab positive in both tests at the respective other examination. This can point towards a very early stage of infection or towards a false-positive result for *T*. *solium* [33, 34]. A recent study reported reactions of this specific band with *T*. *hydatigena* and *E*. *granulosus* in pigs [63]. Cross-reactions in humans with malignancies like lung cancer or other parasitic diseases (like *T*. *saginata*, *E*. *granulosus*, and potentially *Schistosoma ssp*.) were linked with a positive GP50 band only [33–35].

We would like to point out the present study has several limitations: first, the study population comprised only PWE. We considered PWE as an ideal study population in order to search for TSCT in a setting with no previous epidemiological information available, as there is good evidence that higher TSCT prevalence estimates are found in PWE [6, 7, 9, 64, 65]. Hence, it must be considered that the prevalence of TSCT in the general population is probably lower. Second, the catchment area of this study covered only one of three municipalities of Dar es Salaam. To get a more precise prevalence estimate of TSCT in Dar es Salaam, further studies covering different urban areas, including larger sample sizes, would represent an asset. Third, our study was limited by the inability to include copro-Ag testing in the initial recruitment phase and microscopic or molecular testing of stool samples due to financial restrictions. This would have provided a more complete picture of taeniasis and whether potential cross-reacting parasites among affected PWE were present. In addition, electroencephalography and MRI, which could not be included, again due to financial restrictions, would have been of added value and should be included in future studies focusing on PWE. Fourth, there was only a small number of TSCT positive PWE identified in this study which prevented a sufficiently powered risk analysis. Therefore, regarding statistics, our findings have to be interpreted with caution. Last, questions regarding personal hygiene, travel, eating habits were very limited and partially missing. A more detailed questionnaire and epidemiological follow-up of people with TSCT should also be included in future studies in order to get detailed information about past travel histories and infection modes of individuals with TSCT in urban settings.

Regarding AED treatment of enrolled PWE it has to be considered that the data used in this study is not representative of the treatment compliance of PWE in Dar es Salaam. In our study, all PWE took AED as the recruitment was performed in health centers and dispensaries providing these drugs and dealing with PWE on regular treatment. Hunter et al. described in 2016 a large epilepsy treatment gap (ETG) in rural Tanzania of 40.5% [66]. A review of ETG in African countries reported a mean of 46.8% [67]. Therefore, it must be assumed that a treatment gap is also present in the urban PWE population of Dar es Salaam, but further studies would be required.

### Conclusions

In conclusion, based on the examination of PWE our study demonstrates the presence of TSCT in Dar es Salaam and prevalence of definitive NCC of 1.99% underlining the need of serological and radiological diagnostic capacities as well as training and awareness-raising of medical staff in urban settings. However, we emphasize that the prevalence of CC-Abs and taeniasis-Abs identified in this study is low, 2.65% and 1.66% respectively. This suggests that active *T*. *solium* transmission in Dar es Salaam is likely to play only a very minor role. Infections might mostly be contracted during travel to rural areas, or through contaminated food reaching urban markets and street kitchens in town. Adult worm carriers visiting the town for business or family issues might represent another rare source of *T*. *solium* egg-inflow. Completion of the *T*. *solium* life cycle in Dar es Salaam seems to be unlikely, though not totally impossible. Follow-up studies with a larger number of TSCT positive individuals, including a more detailed risk assessment, as well as species-specific coprological tests, are required in order to investigate this topic further.

## Supporting information

S1 Checklist Completed STROBE checklist for the study `*Taenia solium* cysticercosis and taeniasis in urban settings: Epidemiological evidence from a health-center based study among people with epilepsy in Dar es Salaam, Tanzania´. (PDF)

S1 Document Patient questionnaire used in this study: Protocol for new patient. (PDF)

S1 Table Association of socio-demographic and clinical variables and TSCT in people with epilepsy obtained by univariate logistic regression analysis. (PDF)

S2 Table Distribution of *T*. *solium* cysticercosis and taeniasis among people with epilepsy in the recruitment centers of Kinondoni district (1^st^ examination). (PDF)

## References

[1] World Health Organization (2015) WHO estimates of the global burden of foodborne diseases. Foodborne disease burden epidemiology reference group (2007–2015). https://www.who.int/foodsafety/publications/foodborne_disease/fergreport/en/. Accessed 02 June 2019.

[2] Gonzales I, Rivera JT, Garcia HH, Cysticercosis Working Group in Peru. Pathogenesis of *Taenia solium* taeniasis and cysticercosis. Parasite Immunol. 2016 3; 38(3):136–46. 10.1111/pim.12307 26824681

[3] Del Brutto OH. Neurocysticercosis: A review. ScientificWorldJournal. 2012; 2012:159821 10.1100/2012/159821 22312322 PMC3261519

[4] Winkler AS. Neurocysticercosis in sub-Saharan Africa: a review of prevalence, clinical characteristics, diagnosis, and management. Pathog Glob Health. 2012 9; 106(5):261–74. 10.1179/2047773212Y.0000000047 23265550 PMC4005109

[5] Carabin H, Ndimubanzi PC, Budke CM, Nguyen H, Qian Y, Cowan LD, et al Clinical manifestations associated with neurocysticercosis: A systematic review. PLoS Negl Trop Dis. 2011 5; 5(5):e1152 10.1371/journal.pntd.0001152 21629722 PMC3101170

[6] World Health Organization. (2015). Landscape analysis: management of neurocysticercosis with an emphasis on low- and middle-income countries. World Health Organization. http://www.who.int/iris/handle/10665/152896. Accessed 02 June 2019.

[7] Senanayake N and Román GC. Epidemiology of epilepsy in developing countries. Bull World Health Organ 1993; 71(2):247–58. 8490989 PMC2393447

[8] Winkler AS, Blocher J, Auer H, Gotwald T, Matuja W, Schmutzhard E. Epilepsy and neurocysticercosis in rural Tanzania–an imaging study. Epilepsia. 2009 5; 50(5):987–93. 10.1111/j.1528-1167.2008.01867.x 19054402

[9] Del Brutto OH, Rajshekhar V, White AC Jr, Tsang VC, Nash TE, Takayanagui OM, et al Proposed diagnostic criteria for neurocysticercosis. Neurology. 2001 7; 57(2):177–83. 10.1212/wnl.57.2.177 11480424 PMC2912527

[10] Hunter E, Burton K, Iqbal A, Birchall D, Jackson M, Rogathe J et al Cysticercosis and epilepsy in rural Tanzania: a community-based case-control and imaging study. Trop Med Int Health. 2015 9; 20(9):1171–1179. 10.1111/tmi.12529 25940786 PMC5496663

[11] Mwang’onde BJ, Nkwengulila G, Chacha M. The serological survey for human cysticercosis prevalence in Mbulu district, Tanzania. Advances in Infectious Diseases. 2012 9; 2:62–6. 10.4236/aid.2012.23009

[12] Ngowi HA, Winkler AS, Braae UC, Mdegela RH, Mkupasi EM, Kabululu ML, et al *Taenia solium* taeniosis and cysticercosis literature in Tanzania provides research evidence justification for control: A systematic scoping review. PLoS One 14(6):e0217420 10.1371/journal.pone.0217420 31166983 PMC6550401

[13] Mafojane NA, Appleton CC, Krecek RC, Michael LM, Willingham AL 3rd. The current status of neurocysticercosis in Eastern and Southern Africa. Acta Trop. 2003 6; 87(1):25–33. 10.1016/s0001-706x(03)00052-4 12781375

[14] Braae UC, Magnussen P, Ndawi B, Harrison W, Lekule F, Johansen MV. Effect of repeated mass drug administration with praziquantel and track and treat of taeniosis cases on the prevalence of taeniosis in *Taenia solium* endemic rural communities of Tanzania. Acta Trop. 2017 1; 165:246–251. 10.1016/j.actatropica.2015.10.012 26597324

[15] Mwanjali G, Kihamia C, Kakoko DVC, Lekule F, Ngowi H, Johansen MV, et al Prevalence and risk factors associated with human *Taenia solium* infections in Mbozi district, Mbeya region, Tanzania. PLoS Negl Trop Dis. 2013; 7(3):e2102 10.1371/journal.pntd.0002102 23516650 PMC3597471

[16] Coral-Almeida M, Gabriël S, Abatih EN, Praet N, Benitez W, Dorny P. *Taenia solium* human cysticercosis: a systematic review of sero-epidemiological data from endemic zones around the world. PLoS Negl Trop Dis. 2015 7; 9(7):e0003919 10.1371/journal.pntd.0003919 26147942 PMC4493064

[17] Neiderud CJ. How urbanization affects the epidemiology of emerging infectious diseases. Infect Ecol Epidemiol. 2015 6 24; 5:27060 10.3402/iee.v5.27060 26112265 PMC4481042

[18] Stephens C. Urbanisation: the implications for health. Afr Health. 1996 1; 18(2):14–5. 12346887

[19] Mott KE, Desjeux P, Moncayo A, Ranque P, de Raadt P. Parasitic diseases and urban development. Bull World Health Organ. 1990; 68(6):691–8. 2127380 PMC2393177

[20] National Bureau of Statistics, Ministry of Finance; Office of Chief Government Statistician President’s Office, Finance, Economy and Development Planning. Tanzania Population and Housing Census 2012. Ref. TZA_2012_PHC_v01_M. Population and Housing Census 2012. http://catalog.ihsn.org/index.php/catalog/4618. Accessed 02 June 2019.

[21] Tann vom Hove. The world’s fastest growing cities and urban areas from 2006 to 2020. http://www.citymayors.com/statistics/urban_growth1.html. Accessed 02 June 2019.

[22] National Bureau of Statistics, Agriculture Sample Census Survey 2007/2008, version 1.0 of the public use dataset (Dec 2011), provided by the National Bureau of Statistics. http://www.nbs.go.tz/tnada/index.php/catalog. Accessed 02 June 2019.

[23] Penrose K, de Castro MC, Werema J, Ryan ET. Informal urban settlements and cholera risk in Dar es Salaam, Tanzania. PLoS Negl Trop Dis. 2010 3; 4(3): e631 10.1371/journal.pntd.0000631 20300569 PMC2838775

[24] Fisher RS, Acevedo C, Arzimanoglou A, Bogacz A, Cross JH, Elger CE, et al ILAE official report: a practical clinical definition of epilepsy. Epilepsia. 2014 4; 55(4):475–82. 10.1111/epi.12550 24730690

[25] Del Brutto OH, Nash TE, White AC Jr, Rajshekhar V, Wilkins PP, Singh G, et al Revised diagnostic criteria for neurocysticercosis. J Neurol Sci. 2017 1; 372:202–210. 10.1016/j.jns.2016.11.045 28017213

[26] Nash TE, Del Brutto OH, Butman JA, Corona T, Delgado-Escueta A, Duron RM, et al Calcific neurocysticercosis and epileptogenesis. Neurology. 2004 6; 62(11):1934–8. 10.1212/01.wnl.0000129481.12067.06 15184592 PMC2912520

[27] Winkler AS, Kerschbaumsteiner K, Stelzhammer B, Meindl M, Kaaya J, Schmutzhard E. Prevalence, incidence, and clinical characteristics of epilepsy-a community-based door-to-door study in northern Tanzania. Epilepsia. 2009 10; 50(10):2310–3. 10.1111/j.1528-1167.2009.02184.x 19583783

[28] Winkler A, Schaffert M, Schmutzhard E. Epilepsy in resource poor countries—suggestion of an adjusted classification. Epilepsia. 2007 5; 48(5):1029–30. 10.1111/j.1528-1167.2007.01009_1.x 17509005

[29] World Health Organization. (1993). The ICD-10 classification of mental and behavioural disorders: diagnostic criteria for research. World Health Organization. http://www.who.int/iris/handle/10665/37108. Accessed 02 June 2019.

[30] Tsang VC, Brand JA, Boyer AE. An enzyme-linked immunoelectrotransfer blot assay and glycoprotein antigens for diagnosing human cysticercosis (*Taenia solium*). J Infect Dis. 1989 1; 159(1):50–9. 10.1093/infdis/159.1.50 2909643

[31] Deckers N, Dorny P (2010) Immunodiagnosis of *Taenia solium* taeniosis/cysticercosis. Trends Parasitol. 2010 3; 26(3):137–44. 10.1016/j.pt.2009.12.008 20083438

[32] Noh J, Rodriguez S, Lee Y-M, Handali S, Gonzalez AE, Gilman RH, et al Recombinant protein-and synthetic peptide-based immunoblot test for diagnosis of neurocysticercosis. J Clin Microbiol. 2014 5; 52(5):1429–34. 10.1128/JCM.03260-13 24554747 PMC3993633

[33] Kojic EM, White AC Jr. A positive enzyme-linked immunoelectrotransfer blot assay result for a patient without evidence of cysticercosis. Clin Infect Dis. 2003 1; 36(1):e7–9. 10.1086/344445 12491223

[34] Furrows SJ, McCroddan J, Bligh WJ, Chiodini P. Lack of specificity of a single positive 50-kDa band in the electroimmunotransfer blot (EITB) assay for cysticercosis. Clin Microbiol Infect. 2006 5; 12(5):459–62. 10.1111/j.1469-0691.2006.01381.x 16643523

[35] Hancock K, Pattabhi S, Whitfield FW, Yushak ML, Lane WS, Garcia HH, et al Characterization and cloning of T24, a *Taenia solium* antigen diagnostic for cysticercosis. Mol Biochem Parasitol. 2006 5; 147(1):109–17. 10.1016/j.molbiopara.2006.02.004 16540186

[36] Dorny P, Phiri IK, Vercruysse J, Gabriel S, Willingham AL 3rd, Brandt J, et al A Bayesian approach for estimating values for prevalence and diagnostic test characteristics of porcine cysticercosis. Int J Parasitol. 2004 4; 34(5):569–76. 10.1016/j.ijpara.2003.11.014 15064121

[37] Wilkins PP, Allan JC, Verastegui M, Acosta M, Eason AG, Garcia HH, et al Development of a serologic assay to detect *Taenia solium* taeniasis. Am J Trop Med Hyg. 1999 2; 60(2):199–204. 10.4269/ajtmh.1999.60.199 10072136

[38] Levine MZ, Lewis MM, Rodriquez S, Jimenez JA, Khan A, Lin S, Garcia HH, et al Development of an enzyme-linked immunoelectrotransfer blot (EITB) assay using two baculovirus expressed recombinant antigens for diagnosis of *Taenia solium* taeniasis. J Parasitol. 2007 4; 93(2):409–17. 10.1645/GE-938R.1 17539427

[39] Handali S, Klarman M, Gaspard AN, Noh J, Lee YM, et al Multiantigen print immunoassay for comparison of diagnostic antigens for *Taenia solium* cysticercosis and taeniasis. Clin Vaccine Immunol. 2010 1; 17(1):68–72. 10.1128/CVI.00339-09 19906893 PMC2812080

[40] Guezala MC, Rodriguez S, Zamora H, Garcia HH, Armando E. Gonzalez AE, et al Development of a species-specific coproantigen ELISA for human *Taenia solium* taeniosis. Am J Trop Med Hyg. 2009 9; 81(3):433–7. 19706909

[41] Benjamini Y and Hochberg Y. Controlling the false discovery rate: a practical and powerful approach to multiple testing. J Royal Stat. Soc. 1995; Series B 57: 289–300.

[42] Praet N, Kanobana K, Kabwe C, Maketa V, Lukanu P, Lutumba P, et al *Taenia solium* cysticercosis in the Democratic Republic of Congo: how does pork trade affect the transmission of the parasite? PLoS Negl Trop Dis. 2010 9; 4(9): e817 10.1371/journal.pntd.0000817 20838646 PMC2935392

[43] Kamuyu G, Bottomley C, Mageto J, Lowe B, Wilkins PP, Noh JC, et al Exposure to multiple parasites is associated with the prevalence of active convulsive epilepsy in sub-Saharan Africa. PLoS Negl Trop Dis. 2014 5; 8(5):e2908 10.1371/journal.pntd.0002908 24875312 PMC4038481

[44] Schmidt V, Kositz C, Herbinger KH, Carabin H, Ngowi B, Naman E, et al Association between *Taenia solium* infection and HIV/AIDS in northern Tanzania: a matched cross sectional-study. Infect Dis Poverty. 2016 12; 5(1):111 10.1186/s40249-016-0209-7 27903304 PMC5131417

[45] Mwape KE, Phiri IK, Praet N, Muma JB, Zulu G, Van den Bossche P, de Deken R, et al *Taenia solium* infections in a rural area of Eastern Zambia-a community based study. PLoS Negl Trop Dis. 2012 3; 6(3):e1594 10.1371/journal.pntd.0001594 22479664 PMC3313923

[46] Dermauw V, Dorny P, Braae UC, Devleesschauwer B, Robertson LJ, Saratsis A, et al Epidemiology of *Taenia saginata* taeniosis/cysticercosis: a systematic review of the distribution in southern and eastern Africa. Parasit Vectors. 2018 11; 11(1):578 10.1186/s13071-018-3163-3 30400948 PMC6219070

[47] García HH, Del Brutto OH. Heavy nonencephalitic cerebral cysticercosis in tapeworm carriers. The Cysticercosis Working Group in Perú. Neurology. 1999 10; 53(7):1582–4. 10.1212/wnl.53.7.1582 10534273

[48] Gilman RH, Del Brutto OH, García HH, Martínez M. Prevalence of taeniosis among patients with neurocysticercosis is related to severity of infection. The Cysticercosis Working Group in Perú. Neurology. 2000 10; 55(7):1062. 10.1212/wnl.55.7.1062 11061275

[49] Ernest E, Nonga HE, Kynsieri N, Cleaveland S. A retrospective survey of human hydatidosis based on hospital records during the period 1990–2003 in Ngorongoro, Tanzania. Zoonoses Public Health. 2010 12; 57(7–8):e124–9. 10.1111/j.1863-2378.2009.01297.x 19968843

[50] Said K, Hella J, Knopp S, Nassoro T, Shija N, Aziz F, et al Schistosoma, other helminth infections, and associated risk factors in preschool-aged children in urban Tanzania. PLoS Negl Trop Dis. 2017 11; 11(11):e0006017 10.1371/journal.pntd.0006017 29108003 PMC5697890

[51] Kwiringira J, Atekyereza P, Niwagaba C, Kabumbuli R, Rwabukwali C, Kulabako R, et al Seasonal variations and shared latrine cleaning practices in the slums of Kampala city, Uganda. BMC Public Health. 2016 4; 16:361 10.1186/s12889-016-3036-7 27121388 PMC4847375

[52] Kimani-Murage EW, Ngindu AM. Quality of water the slum dwellers use: the case of a Kenyan slum. J Urban Health. 2007 11; 84(6): 829–38. 10.1007/s11524-007-9199-x 17551841 PMC2134844

[53] Tumwine J, Thompson J, Katui-Katua M, Mujwahuzi M, Johnstone N, Porras I. Sanitation and hygiene in urban and rural households in East Africa. Int J Environ Health Res. 2003 6; 13(2):107–15. 10.1080/0960312031000098035 12745333

[54] Secka A., Grimm F, Victor B, Marcotty T, De Deken R, Nyan O, et al Epilepsy is not caused by cysticercosis in The Gambia. Trop Med Int Health. 2010 4; 15(4):476–9. 10.1111/j.1365-3156.2010.02470.x 20180937

[55] Ndimubanzi PC, Carabin H, Budke CM, Nguyen H, Qian YJ, Rainwater E, et al A systematic review of the frequency of neurocysticercosis with a focus on people with epilepsy. PLoS Negl Trop Dis. 2010 11; 4(11):e870 10.1371/journal.pntd.0000870 21072231 PMC2970544

[56] Millogo A, Nitiéma P, Carabin H, Boncoeur-Martel MP, Rajshekhar V, Tarnagda Z, et al Prevalence of neurocysticercosis among people with epilepsy in rural areas of Burkina Faso. Epilepsia. 2012 12; 53(12):2194–202. 10.1111/j.1528-1167.2012.03687.x 23148555 PMC5459323

[57] Mwape KE, Blocher J, Wiefek J, Schmidt K, Dorny P, Praet N, et al Prevalence of neurocysticercosis in people with epilepsy in the Eastern Province of Zambia. PLoS Negl Trop Dis. 2015 8; 9(8):e0003972 10.1371/journal.pntd.0003972 26285031 PMC4540454

[58] Bruno E, Bartoloni A, Zammarchi L, Strohmeyer M, Bartalesi F, Bustos JA, et al Epilepsy and Neurocysticercosis in Latin America: A Systematic Review and Meta-analysis. PLoS Negl Trop Dis. 2013 10; 7(10):e2480 10.1371/journal.pntd.0002480 24205415 PMC3814340

[59] Rottbeck R, Nshimiyimana JF, Tugirimana P, Düll UE, Sattler J, Hategekimana JC, et al High prevalence of cysticercosis in people with epilepsy in southern Rwanda. PLoS Negl Trop Dis. 2013 11; 7(11):e2558 10.1371/journal.pntd.0002558 24244783 PMC3828157

[60] Singh G, Singh P, Singh I, Rani A, Kaushal S, Avasthi G. Epidemiologic classification of seizures associated with neurocysticercosis: observations from a sample of seizure disorders in neurologic care in India. Acta Neurol Scand. 2006 4; 113(4):233–40. 10.1111/j.1600-0404.2005.00575.x 16542162

[61] Prasad KN, Prasad A, Gupta RK, Nath K, Pradhan S, Tripathi M, et al Neurocysticercosis in patients with active epilepsy from the pig farming community of Lucknow district, north India. Trans R Soc Trop Med Hyg. 2009 2; 103(2):144–50. 10.1016/j.trstmh.2008.07.015 18804830

[62] Moyano LM, Saito M, Montano SM, Gonzalvez G, Olaya S, Ayvar V, et al Neurocysticercosis as a cause of epilepsy and seizures in two community-based studies in a cysticercosis-endemic region in Peru. PLoS Negl Trop Dis. 2014 2; 8(2): e2692 10.1371/journal.pntd.0002692 24551255 PMC3923674

[63] Gomez-Puerta L, Vargas-Calla A, Castillo Y, Lopez-Urbina MT, Dorny P, Garcia HH, et al Evaluation of cross-reactivity to *Taenia hydatigena* and *Echinococcus granulosus* in the enzyme-linked immunoelectrotransfer blot assay for the diagnosis of porcine cysticercosis. Parasit Vectors. 2019 1; 12(1):57 10.1186/s13071-018-3279-5 30678700 PMC6346519

[64] Ngugi AK, Bottomley C, Kleinschmidt I, Wagner RG, Kakooza-Mwesige A, Ae-Ngibise K, et al Prevalence of active convulsive epilepsy in sub-Saharan Africa and associated risk factors: cross-sectional and case-control studies. Lancet Neurol. 2013 3; 12(3):253–63. 10.1016/S1474-4422(13)70003-6 23375964 PMC3581814

[65] Singh G, Bawa J, Chinna D, Chaudhary A, Saggar K, Modi M, et al Association between epilepsy and cysticercosis and toxocariasis: a population-based case-control study in a slum in India. Epilepsia. 2012 12; 53(12):2203–8. 10.1111/epi.12005 23106145

[66] Hunter E, Rogathi J, Chigudu S, Jusabani A, Jackson M, Whittaker RG, et al The epilepsy treatment gap in rural Tanzania: A community-based study in adults. Seizure. 2016 3; 36:49–56. 10.1016/j.seizure.2016.02.008 26938970

[67] Mbuba CK, Ngugi AK, Newton CR, Carter JA. The epilepsy treatment gap in developing countries: a systematic review of the magnitude, causes, and intervention strategies. Epilepsia. 2008 9; 49(9):1491–503. 10.1111/j.1528-1167.2008.01693.x 18557778 PMC3573323

