# Supplementary material for: Taenia solium cysticercosis and taeniasis in urban settings: Epidemiological evidence from a health-center based study among people with epilepsy in Dar es Salaam, Tanzania
Source: PLoS Negl Trop Dis. 2019 Dec 6;13(12):e0007751. doi: 10.1371/journal.pntd.0007751 (PMC6897529; doi:10.1371/journal.pntd.0007751)
Supplement: S1 Checklist — (PDF) [file pntd.0007751.s001.pdf]

STROBE checklist.

	Item No.	Recommendation	Section/paragraph where indicated
**Title and abstract**			
	1	(*a*) Indicate the study's design with a commonly used term in the title or the abstract	Indicated in the title
		(*b*) Provide in the abstract an informative and balanced summary of what was done and what was found	Indicated in the abstract
**Introduction**			
Background/rationale	2	Explain the scientific background and rationale for the investigation being reported	Explanations provided in the Background section
Objectives	3	State specific objectives, including any prespecified hypotheses	Main objectives mentioned at the end of the Background section
**Methods**			
Study design	4	Present key elements of study design early in the paper	Presented in the Methods section in the subsection `Enrollment of study participants´ and shown in Figure 2
Setting	5	Describe the setting, locations, and relevant dates, including periods of recruitment, exposure, follow-up, and data collection	Described throughout Methods section

Participants	6	(*a*) *Cohort study*—Give the eligibility criteria, and the sources and methods of selection of participants. Describe methods of follow-up<br>*Case-control study*—Give the eligibility criteria, and the sources and methods of case ascertainment and control selection. Give the rationale for the choice of cases and controls<br>*Cross-sectional study*—Give the eligibility criteria, and the sources and methods of selection of participants	NA<br>NA<br>Described in the Methods section in the subsection `Enrollment of study participants´
		(*b*) *Cohort study*—For matched studies, give matching criteria and number of exposed and unexposed<br>*Case-control study*—For matched studies, give matching criteria and the number of controls per case	NA<br>NA
Variables	7	Clearly define all outcomes, exposures, predictors, potential confounders, and effect modifiers. Give diagnostic criteria, if applicable	Described in the Methods section in the subsection `Interview and neurological examination´
Data sources/ measurement	8*	For each variable of interest, give sources of data and details of methods of assessment (measurement). Describe comparability of assessment methods if there is more than one group	Described in the Methods section in according subsections
Bias	9	Describe any efforts to address potential sources of bias	Described in the Methods section in according subsections
Study size	10	Explain how the study size was arrived at	Described in the Methods section in according subsections
Quantitative variables	11	Explain how quantitative variables were handled in the analyses. If applicable, describe which groupings were chosen and why	Described in the Methods section in the subsection `Data analysis´
Statistical methods	12	(a) Describe all statistical methods, including those used to control for confounding	Described in the Methods section in the subsection `Data analysis´

		(b) Describe any methods used to examine subgroups and interactions	NA
		(c) Explain how missing data were addressed	Described in the Methods section and presented in Table 1 and 2
		(d) Cohort study—If applicable, explain how loss to follow-up was addressed	Shown in Table 1 and 2
		Case-control study—If applicable, explain how matching of cases and controls was addressed	NA
		Cross-sectional study—If applicable, describe analytical methods taking account of sampling strategy	NA
		(e) Describe any sensitivity analyses	Described in the Methods section in the subsection \`Data analysis´
**Results**			
Participants	13*	(a) Report numbers of individuals at each stage of study—eg numbers potentially eligible, examined for eligibility, confirmed eligible, included in the study, completing follow-up, and analysed	Reported in Results section and shown in Figure 2
		(b) Give reasons for non-participation at each stage	Indicated in the results section and in the regarding sub-sections
		(c) Consider use of a flow diagram	Included as Figure 2
Descriptive data	14*	(a) Give characteristics of study participants (eg demographic, clinical, social) and information on exposures and potential confounders	Described in the Results section in the subsection \`Socio-demographic baseline data´ and presented in Table 1
		(b) Indicate number of participants with missing data for each variable of interest	Indicated in Table 1 and 2

		(c) Cohort study—Summarise follow-up time (eg, average and total amount)	NA
Outcome data	15*	Cohort study—Report numbers of outcome events or summary measures over time	NA
		Case-control study—Report numbers in each exposure category, or summary measures of exposure	NA
		Cross-sectional study—Report numbers of outcome events or summary measures	Described in the Results section and regarding subsections
Main results	16	(a) Give unadjusted estimates and, if applicable, confounder-adjusted estimates and their precision (eg, 95% confidence interval). Make clear which confounders were adjusted for and why they were included	NA
		(b) Report category boundaries when continuous variables were categorized	Reported in the Results section and in Table 1 and 2
		(c) If relevant, consider translating estimates of relative risk into absolute risk for a meaningful time period	NA
Other analyses	17	Report other analyses done—e.g. analyses of subgroups and interactions, and sensitivity analyses	NA
**Discussion**			
Key results	18	Summarise key results with reference to study objectives	Summarized in the first half of the Discussion section
Limitations	19	Discuss limitations of the study, taking into account sources of potential bias or imprecision. Discuss both direction and magnitude of any potential bias	Summarized in the second half of the Discussion section

Interpretation	20	Give a cautious overall interpretation of results considering objectives, limitations, multiplicity of analyses, results from similar studies, and other relevant evidence	Provided throughout the Discussion section
Generalisability	21	Discuss the generalisability (external validity) of the study results	Provided in the last paragraph of the Discussion section
**Other information**			
Funding	22	Give the source of funding and the role of the funders for the present study and, if applicable, for the original study on which the present article is based	Provided in the Financial disclosure and competing interest statements in the online submission form

NA: Not Applicable.

*Give information separately for cases and controls in case-control studies and, if applicable, for exposed and unexposed groups in cohort and cross-sectional studies.

Note: An Explanation and Elaboration article discusses each checklist item and gives methodological background and published examples of transparent reporting. The STROBE checklist is best used in conjunction with this article (freely available on the Web sites of PLoS Medicine at http://www.plosmedicine.org/, Annals of Internal Medicine at http://www.annals.org/, and Epidemiology at http://www.epidem.com/). Information on the STROBE Initiative is available at www.strobe-statement.org.

Source of this template:
STROBE checklist for cohort, case-control, and cross-sectional studies. Available: http://www.strobe-statement.org/index.php?id=available-checklist. Accessed 18 March 2018.
