# Supplementary material for: Taenia solium cysticercosis and taeniasis in urban settings: Epidemiological evidence from a health-center based study among people with epilepsy in Dar es Salaam, Tanzania
Source: PLoS Negl Trop Dis. 2019 Dec 6;13(12):e0007751. doi: 10.1371/journal.pntd.0007751 (PMC6897529; doi:10.1371/journal.pntd.0007751)
Supplement: S1 Document — (PDF) [file pntd.0007751.s002.pdf]

Protocol for New Patients

1. Demographics:

Date: ____

Name:____

Patient (Pt) number:______________ Date of birth:____________________

Gender: ____

Tribe: ____

Religion: ____

Occupation: ____

Marital status: ____________________ Age when married: ________________

N° of children: ____

Head of household: ____

Ten-Cell leader: ____

Phone number of the Pt: ____

Address of the Pt: ____

How long has Pt been living in Dar es Salaam? ____________________________

Contact person: ____________________ Phone number: ____________________

Witness (name and relation to patient):______________________________

Phone number of witness: ____

Health (recruitment) center: ____

Distance to health center: _____________ Means of transport: ________________

Duration: ____

2. Pork consumption:

Do you eat pork? □ Yes □ No

Anybody in your family eating pork? □ Yes □ No

Are there free roaming pigs around? □ Yes □ No

Have you or any family member ever had a pork tapeworm? □ Yes□ No

3. Anthelmintic treatment during last 12 months?

□ Yes □ No □ Unkown

4. Description of epileptic seizure(s):

4.1 Description by Pt and witness first in words (step by step):

____

____

4.2 Loss of consciousness:

□ From the beginning □ After motor signs start □ After aura

□ Clouding of consciousness □ No loss

4.3 Motor activity:

□ Tonic □ Clonic □ Both

□ No movements of limbs, but rolling of eyes and grinding of teeth

□ No movements at all

4.4 Side of limb movements:

□ Only left side □ Only right side □ Both sides

4.5 Fitting for how long? ____

Ever fitted >than 30 min? □ Yes □ No

If yes, give time? ____

4.6 Supportive signs:

□ Froth from mouth □ Tongue/lip bite

□ Urine/faecal incontinence

4.7 Contracted any injuries during fits? □ Yes □ No

What kind of injury (burn, bruise etc.?, where?, size in cm?)_______________

____

When contracted? ____

Medical attention sought? □ Yes □ No

Where? □ Hospital □ Dispensary □ Traditional healer

Action taken? □ Surgery □ Dressing

□ Drugs, which? ________________________________

□ Other: ____

4.8 Reorientation (postictal) phase present? □ Yes □ No

How long? ____

Description of reorientation phase: ____________________________________

____

□ Aggression □ Confusion □ Sleeping/sleepy

4.9 Are epileptic seizures always the same? □ Yes □ No

If different, describe and make clear to which type of seizure(s) the information in the questionnaire refers to? ______________________________

____

5. Frequency:

When did seizures start?

Date: ____________________ Last seizure: ___________________________

How many seizures in total: ____

How many seizures within one month: ______________________________________

Does the Pt have multiple seizures within one day (how many): __________________

If yes, is Pt waking up in between? ____________________________________

What was Pt doing just before first seizure? __________________________________

Was Pt healthy just before the first seizures? ______________________________

When during the day are most of the seizures?

□ Waking or the hour thereafter □ Morning □ Afternoon

□ Anytime during the day □ Evening □ Night while asleep

□ Anytime at day or night □ Other:________________________

6. Prodromi/Aura:

□ Seizures start suddenly □ Pt knows when they are coming (premonition)

Description of sensation: ____

____

How long before? ____

Focal signs like twitching of arm/leg before losing consciousness?

□ Yes □ No

How long before? ____

7. Association of other illnesses in relation to first epileptic seizure:

Any illness prior to first seizure □ Yes □ No

Details (what illness, how long before first seizure): _____________________

____

8. Precipitants: □ Yes □ No

Any precipitants that bring seizures on?

□ Fever □ Alcohol □ Menstruation □ Sleep

□ Emotional stimuli □ Other:________________________________

9. Past medical history (before the seizures started):

9.1 Description of **severe/chronic illnesses** in the past (type, date, action taken, permanent condition): ____

____

9.2 Does/did the Pt drink **alcohol** (age when started, age when stopped, how many days per week, how much does Pt drink, how often does Pt get drunk):____________

____

9.3 Febrile fits (FF) in the past (how many episodes, age, how many fits per episode per 24 hrs, FF unilateral/bilateral, average time of FF, sequelae):

____

____

9.4 Accidents, injuries or traumata (type, date, action taken, permanent condition):

____

____

9.5 Hospital admissions (what hospital, reason, date, time of stay):

____

____

10. Episodes of severe progressive headache within last months?

□ Yes □ No □ Unkown

11. Past psychiatric history: □ Yes □ No

Description of psychiatric problems in the past and at present:

□ Depression □ Mental retardation □ Behavioural problems

□ Dementia □ Psychotic episodes

□ Other:____

Severity: □ Mild □ Moderate □ Severe

Since when: ____

Action taken: ____

12. Drug history:

12.1 Pt currently on treatment (Tx): □ Yes □ No

Details: ____

12.2 Has the Pt ever been on AED? □ Yes □ No

Details (drugs, dosage, how long): ____________________________________

____

12.3 Herbal Tx tried? □ Yes □ No

Description (what ingredient, route, when started, for how long taken, any help, side effects): ____

____

12.4 Scarifications performed? □ Yes □ No

Description (when, where, manipulation of wound, any help, side effects):

____

____

13. Family history:

Any known cases of epilepsy/seizure(s) in family? □ Yes □ No

Who? ____

Description of seizure(s) (age at first, last seizure, frequency, precipitant(s)):

____

____

Any known cases of other neurological/mental illnesses in family?

□ Yes □ No

Who? ____

What illness? ____

14. Birth history and perinatal history:

14.1 Was **mother** healthy during pregnancy?□ Yes □ No

Type of illness, medication taken: ____________________________________

14.2 Description of delivery:

□ Hospital □ Dispensary □ Home

□ On term □ Preterm □Spontaneous vaginal delivery□ Caesarean section □ Assisted delivery (forceps etc.)

14.3 Labour:

□ Prolonged □ Precipitated □ Normal

Duration in hours: ____

14.4 Baby:

Baby crying soon? □ Yes □ No

Baby sucking well? □ Yes □ No

Baby blue? □ Yes □ No

Baby yellow? □ Yes □ No

14.5 Other problems (resuscitation, incubator, oxygen supply, **neonatal seizures** etc.):

____

14.6 Milestones:

Delayed/normal?

Head control	Turning	Sitting	Crawling	Standing	Walking	Talking

Delayed since when: ________________________________since birth

Special event: ____

14.7 Neonatal seizures (age, frequency, any associated symptoms/signs): _________

____

15. School attendance:

Did/does Pt go to school? □ Yes

□ No, reason: ________________________________

Age started?

____

Level achieved?

____

Regular attendance? □ Yes

□ No, reason:

□ Epilepsy □ Financial □ Social

□ Other: ____________________________________

Drop out? □ Yes

□ No, reason:

□ Epilepsy □ Financial □ Social

□ Other: ____

16. Work:

Before first fit: Type of work? ______________________________________

Hours of work? ______________________________

Since fits started: Type of work? ______________________________

Hours of work? ______________________________

Since Tx started: Type of work? ______________________________

Hours of work? ______________________________

If there is a difference, give reason: ______________________________

After a fit: Days lost due to fits: ______________________________

Days with impaired work: ______________________________

On days with impaired work Pt works: □ < 50% □ > 50%

17. Neurological signs:

____

____

____

18. Mental state:

Appearance: □ Normal □ Abnormal: ______________________

Behaviour: □ Normal □ Abnormal: ______________________

Cooperation: □ Normal □ Abnormal: ______________________

Affect: □ Normal □ Abnormal: ______________________

Speech: □ Normal □ Abnormal: ______________________

Mood: □ Normal □ Abnormal: ______________________

Thought: □ Normal □ Abnormal: ______________________

Cognition: □ Normal □ Abnormal: ______________________

19. Impression:

19.1 Kind of seizures:

□ Symptomatic (provoked) seizures:

Cause: □ Alcohol □ Fever □ Malaria

□ Other: ______________________________

□ Febrile seizures

□ Unprovoked single seizure

□ Pseudoseizures

19.2 Type of epileptic seizures (see explanatory notes)**:**

□ Generalized seizures within a specific age range

□ Generalized seizures outside a specific age range

□ Generalized seizures with brain damage

□ Generalized seizures with focal signs

□ Simple partial seizures

□ Two different seizure types, which? ______________________________

□ Unclassified seizures

□ Other types of seizures (juvenile myoclonic, Salaam attacks, absences, complex partial): ____

19.3 Loss of consciousness only:

□ Provoked, cause: ____

□ Unprovoked

19.4 Syncope and pre-syncope: □ Yes □ No

19.5 Non-epileptic myoclonus: □ Yes □ No

19.6 Psychiatric diagnosis:

□ Yes, specify: ____

□ No

20. Plan:

□ Phenobarbitone	Dose:_____________	Tabs given:_________________
□ Carbamazepine	Dose:_____________	Tabs given:_________________
□ Valproate	Dose:_____________	Tabs given:_________________

Health education done? □ Yes □ No

Next appointment: ____

Comments:____

____

____

____

<u>**Explanatory notes to types of epileptic seizures:**</u>

1. Generalized types of seizure:

a. Generalized seizures within a specific age range:
= primary generalized seizures that start within a specific age group (mainly between 6 and 25 years old). Seizures in this group can also be termed idiopathic generalized epilepsy.

Cause: none obvious
Prognosis: good
Investigations: keep to a minimum

b. Generalized seizures outside a specific age range:
= primary generalized seizures that start outside the specific age range of most of the idiopathic generalized epilepsies where no symptoms/signs on clinical examination suggest a secondary cause.
Cause: none obvious, seizures may be "cryptogenic"
Prognosis: variable
Investigations: further investigations necessary (EEG, neuroimaging)

2. Partial types of seizure that clinically appear as generalized seizures:

a. Generalized seizures with diffuse brain damage:
= in clinical terms these seizures start in a generalized way but diffuse brain damage with mental retardation is obvious. The large majority of patients are children.
Cause: mostly known (e.g. birth defect)
Prognosis: variable
Investigations: keep to a minimum

b. Generalized seizures with focal signs:
= secondary generalized seizures with a focal start, clear unilateral seizures or focal neurological signs upon examination but without major brain damage.
Cause: becomes obvious upon further investigation
Prognosis: depends on the underlying cause
Investigations: further investigations necessary (EEG, neuroimaging)

Reference:

Winkler A, Schaffert M, Schmutzhard E (2007) Epilepsy in resource poor countries - suggestion of an adjusted classification. Epilepsia 48(5):1029-30.
