# Supplementary material for: Taenia solium cysticercosis and taeniasis in urban settings: Epidemiological evidence from a health-center based study among people with epilepsy in Dar es Salaam, Tanzania
Source: PLoS Negl Trop Dis. 2019 Dec 6;13(12):e0007751. doi: 10.1371/journal.pntd.0007751 (PMC6897529; doi:10.1371/journal.pntd.0007751)
Supplement: S1 Table — (PDF) [file pntd.0007751.s003.pdf]

S1 Table. Association of socio-demographic and clinical variables and TSCT in people with epilepsy obtained by univariate logistic regression analysis.

Variables	*p-value*	Odds ratio	95% CI of Odds
Sex	0.71	1.32	0.31 - 5.71
Age	0.24	0.85	0.65 - 1.11
School education	0.40	0.74	0.37 - 1.48
Religion	0.92	1.21	0.03 - 42.90
Occupation	0.98	1.00	0.75 - 1.35
Period of residency in Dar es Salaam	0.74	1.17	0.46 - 3.01
Pork consumption	0.99	0.98	0.03 - 38.38
Family eats pork	0.80	0.75	0.08 - 7.04
Latrine usage	0.52	2.29	0.18 - 28.75
Took any anthelmintic drug in the past twelve months	0.33	0.32	0.03 - 3.13
Chronic progressive headaches	0.56	0.58	0.09 - 3.59
Age at first seizure	0.06 *	0.94	0.88 - 1.00
Frequency of seizures per month before treatment	0.74	1.00	0.97 - 1.05
Type of seizures	0.58	0.81	0.38 - 1.71
Motor activity during seizures	0.86	0.90	0.28 - 2.91
Aura present before seizures	0.04*	11.60	1.14 - 118.06
Psychiatric illness	0.83	1.20	0.23 - 6.22

CI: confidence interval.
