# Supplementary material for: Taenia solium cysticercosis and taeniasis in urban settings: Epidemiological evidence from a health-center based study among people with epilepsy in Dar es Salaam, Tanzania
Source: PLoS Negl Trop Dis. 2019 Dec 6;13(12):e0007751. doi: 10.1371/journal.pntd.0007751 (PMC6897529; doi:10.1371/journal.pntd.0007751)
Supplement: S2 Table — (PDF) [file pntd.0007751.s004.pdf]

S2 Table. Distribution of *T. solium* cysticercosis and taeniasis among people with epilepsy in the recruitment centers of Kinondoni district (1st examination).

Variables	Recruited PWE		PWE with NCC		PWE with CC-Ag		PWE with CC-Abs		PWE with taeniasis-Abs	
	n	%	n	%	n	%	n	%	n	%
Total	302	100	5	1.66	3	0.99	8	2.65	2	0.66
Magomeni Hospital	108	35.76	2	0.66	0	0	3	0.99	0	0
Tandale Dispensary	26	8.61	0	0	0	0	0	0	0	0
Kiluvya Health Center	10	3.31	0	0	0	0	0	0	0	0
Mwananyamala Hospital	88	29.14	1	0.33	2	0.66	3	0.99	1	0.33
Sinza Hospital	23	7.62	0	0	0	0	0	0	0	0
Kimara Dispensary	47	15.56	2	0.66	1	0.33	2	0.66	1	0.33

PWE: people with epilepsy; NCC: neurocysticercosis; CC: cysticercosis; Ag: antigen; Abs: antibodies; n: number.
